# TLR2 activation induces antioxidant defence in human monocyte-macrophage cell line models

**DOI:** 10.18632/oncotarget.17342

**Published:** 2017-04-21

**Authors:** Iwona Karwaciak, Michal Gorzkiewicz, Grzegorz Bartosz, Lukasz Pulaski

**Affiliations:** ^1^ Laboratory of Transcriptional Regulation, Institute of Medical Biology PAS, Lodz, Poland; ^2^ Department of Molecular Biophysics, Faculty of Biology and Environmental Sciences, University of Lodz, Lodz, Poland; ^3^ Department of General Biophysics, Faculty of Biology and Environmental Sciences, University of Lodz, Lodz, Poland

**Keywords:** monocyte, macrophage, redox homeostasis, differentiation, pattern recognition receptor

## Abstract

When monocytes are recruited to inflammation/infection sites, extravasate and differentiate into macrophages, they encounter increasing levels of oxidative stress, both from exogenous and endogenous sources. In this study, we aimed to determine whether there are specific biochemical mechanisms responsible for an increase in oxidative stress resistance in differentiating macrophages. We performed experiments on *in vitro* cell line models of the monocyte-macrophage differentiation axis (less differentiated THP-1 cells and more differentiated Mono Mac 6 cells). At the same time, we verified the hypothesis that activating monocyte/macrophage innate immune response by pathogens (exemplified by stimulating the TLR2 pattern recognition receptor) would further strengthen cellular antioxidant defences. We found that resistance to exogenous oxidative stress increased substantially both during differentiation and upon activation of TLR2. This increase in antioxidant resistance was accompanied by decrease in free radical damage to cellular proteins. On the molecular level, this resistance was mediated especially by increased levels and activity of glutathione, glutathione-related antioxidant enzymes and Mn superoxide dismutase, as shown by gene expression assays, Western blotting and enzyme activity assays. Moreover, upon TLR2 activation additional molecular mechanisms came into play, conferring additional resistance levels even upon differentiated macrophage-like cells, mainly related to thioredoxin-linked antioxidant enzymes.

## INTRODUCTION

Monocytes are a pivotal element of the innate immune system, circulating in blood and quickly reacting to infection and/or inflammatory challenge by moving by chemotaxis to the affected site, migrating from the bloodstream into tissues and differentiating into macrophages which are powerful early-response immune effectors [1]. Monocytes also have direct regulatory and effector functions in immune defence, independent of their differentiation capacity [2, 3]. The monocyte-macrophage differentiation branch of the myeloid hematopoietic lineage should be understood as a continuum of changing mechanisms and responses, all adapted to efficient reaction to infection.

*In vitro* cultured cell lines, a mainstay of modern experimental biology, are especially helpful for investigating basic biochemical and genetic mechanisms in a relatively isolated and well-characterised, but still physiologically relevant setting. Therefore, they are commonly used in studies on the impact of external factors on cellular homeostatic mechanisms, including redox homeostasis, the delicate balance between pro-oxidant and anti-oxidant activities that ensures not only survival of oxidatively respiring cells, but also robust resistance to environmental oxidative stress [4–6]. Specifically, the availability of immortal, clonal cell lines of the monocytic lineage made it possible to study monocyte and macrophage function in molecular detail. Among the most commonly used and physiologically relevant models of this type, the THP-1 cell line [7] is a gold standard for studying early stages of monocyte differentiation, while the more mature Mono Mac 6 cell line [8] allows the study of mechanisms emerging in more developed macrophages.

Oxidative stress is prevalent in the innate immune system, derived both from endogenous sources (oxidative burst in immune cells) and the cellular microenvironment (enhanced reactive oxidant production at infection and/or inflammation sites). Since this oxidative response is central to efficient anti-microbial action and reactive oxidants are important direct toxins against infectious microorganisms, the presence of oxidative stress must be considered physiological for immune cells, especially macrophages which must be present at the very site of immune response [9–12]. Therefore, antioxidant resistance is crucial for survival and correct function of monocytes and macrophages, and their redox homeostasis is known to be both robust and tightly regulated, although molecular mechanisms of this regulation are still obscure [13–14].

Redox homeostasis in mammalian cells is mediated mainly by a number of enzymatic and non-enzymatic mechanisms for removal of potentially dangerous reactive oxidant molecules. While the level of many small-molecule, cell-permeable antioxidants (e.g. ascorbate or vitamin E) is regulated predominantly at the level of whole organism, each individual cell autonomously regulates the expression of intracellular antioxidant enzymes and peptide (thiol) antoxidants [15]. Among the thiol antioxidants, some are genetically expressed (thioredoxin) and some are biochemically synthesised (glutathione), but all exert their function with help of a plethora of accessory enzymes (reductases, peroxidases etc.), which together form distinct antioxidant systems to facilitate safe electron transfer [16–17].

While it is expected that redox homeostasis evolves together with changing cell fate during differentiation of monocytes and macrophages, it is important to assess this phenomenon also with regard to actual immune activity, i.e. functional activation of both monocytes and macrophages upon stimulation for immune response. In innate immunity, the central triggering mechanism for cellular activation are pattern recognition receptors, especially from the Toll-like receptor (TLR) family [18, 19]. The impact of TLR signalling on redox homeostasis is acknowledged in various cell types on the phenotype level, but it is sometimes difficult to directly identify the molecular mechanisms responsible for enhanced resistance to oxidants [20, 21].

One of the most important TLR family members is TLR2, a pattern recognition receptor for bacterial lipoproteins and lipopeptides. It is expressed at relatively high levels on the surface of monocytes and macrophages [22] and mediates a large number of mostly proinflammatory interactions between microbial components and the innate immune system. The interaction of pathogens with TLR2 results in activation of NF-κB and release of IL-1, IL-6, IL-8, IL-10, IL-12p40, TNF-α and nitric oxide from human monocytes and macrophages [23–26]. TLR2 stimulation induces the expression of phagocytic receptors and results in enhanced phagocytosis of bacteria by macrophages [27]. TLR2 activity is crucial e.g. for *Mycobacterium tuberculosis*-mediated macrophage death [28] or *Porphyromonas gingivalis*-induced oral bone loss upon macrophage activation [29], while the cellular effects of its activation in the monocyte/macrophage lineage, primarily mediated by the NFκB pathway, include induction of Fcγ expression and activity [30]. Also for oncogenesis, TLR2-mediated signalling was shown to play important roles in tumorigenesis [31], since macrophages are involved in clearance of damaged tissues and dead cells, but once clearance has been completed, the persistent activation of inflammatory cells, including elevated ROS production and oxidative stress, can further aid tumour progression, promotion and metastasis.

We decided to use available *in vitro* cell line models of the monocyte-macrophage differentiation axis to study the evolution of redox homeostasis mechanisms along this axis, but also to verify the capability of these mechanisms to react to infectious challenge (in the form of activation of TLR2) at various points along the differentiation continuum. At the basis of our experimental design is an orthogonal approach to differentiation and activation: we compare the response to TLR2 ligand in undifferentiated and differentiated cell types, evaluating antioxidant mechanisms and phenotypes at all stages.

## RESULTS

### Activation of TLR2 increases the resistance of monocyte/macrophage cell lines to oxidative stress-mediated cell death

In order to compare the effect of TLR2 activation on redox homeostasis in models of the monocyte/macrophage lineage at various stages of differentiation, we applied two well-characterised cell line models of *in vitro* macrophage-like differentiation. While the THP-1 cell line is relatively weakly differentiated in the basal state and acquires only some macrophage-like features upon PMA-induced differentiation, the more recently derived Mono Mac 6 cell line shows many monocyte hallmarks already in the basal state and upon vitamin D3/TGFβ challenge displays a complex transition mimicking the macrophage phenotype quite closely. First, we compared the antioxidant defence phenotype (cellular survival after pro-oxidant exposure) of both cell lines in the undifferentiated and differentiated state by challenging them with reactive oxidant-generating compounds known to induce oxidative stress by different mechanisms in cells cultured *in vitro*. Hydrogen peroxide is a broad spectrum reactive oxygen species which can be transformed to other, more reactive species in a biological matrix through several chemical pathways; mixture of ascorbate and ferrous ions efficiently generates highly reactive hydroxyl radicals; paraquat undergoes redox cycling transferring electrons (e.g. from NADPH) onto molecular oxygen, forming the superoxide anion radical.

**Figure 1:**
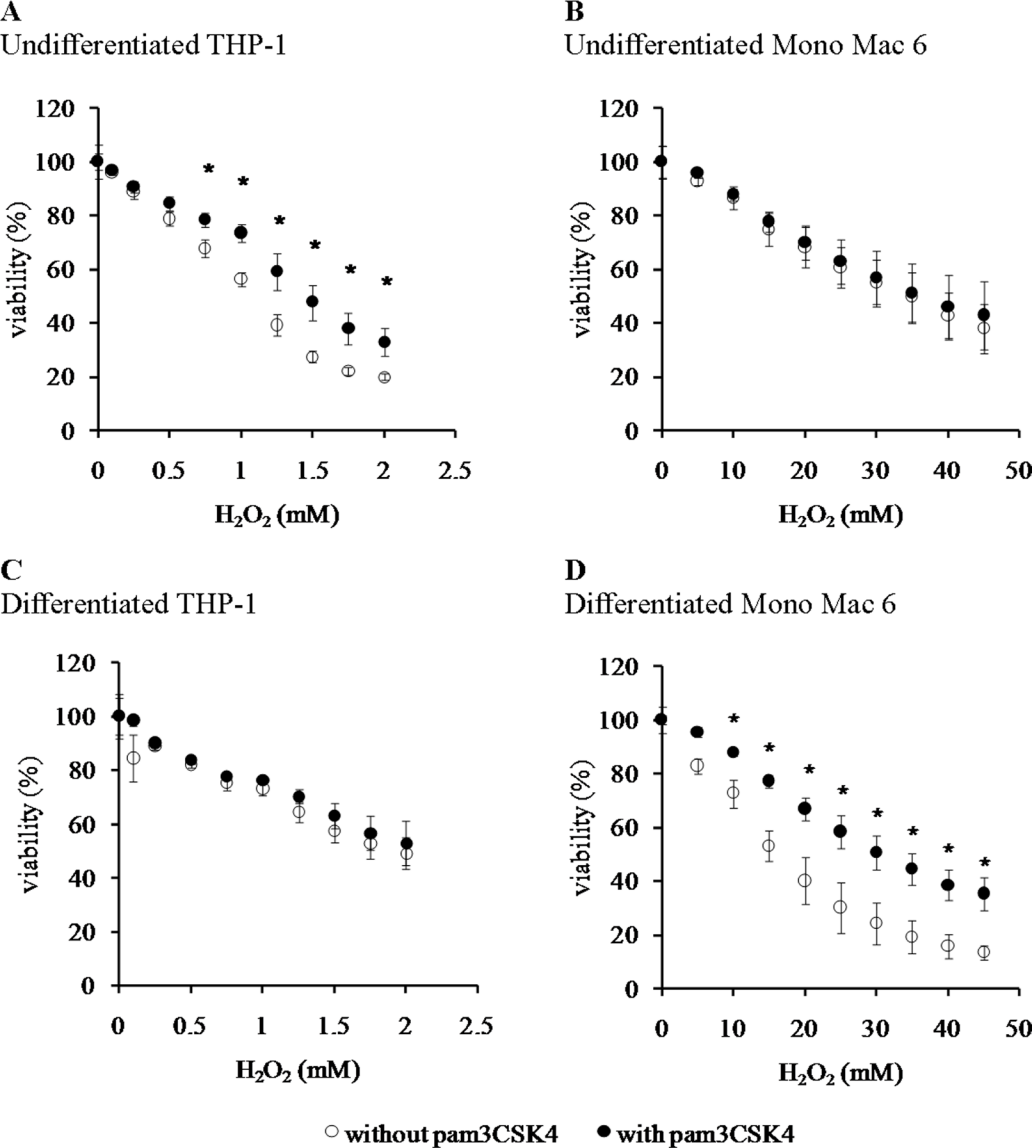
Viability of monocyte/macrophage cell lines upon exposure to hydrogen peroxide THP-1 (panels **A**, **C**) and Mono Mac 6 (panels **B**, **D**) cells were assayed without differentiation (panels A, B) or after differentiation as described in Materials and Methods (panels C, D). Undifferentiated and differentiated cells were subsequently untreated (empty circles) or treated with 500 ng/ml pam3CSK4 (for TLR2 simulation) for 24 h (filled circles). Subsequently, cells were exposed to indicated concentrations of hydrogen peroxide for 6 h and their viability was assayed with the resazurin method. Data presented as mean ± S.E.M. of percentage of viability in control (not exposed to oxidant) sample, *n* = 5 (A, C) or *n* = 4 (B, D). Asterisks indicate statistically significant difference between the TLR2-stimulated and unstimulated cells, *p <* 0.05.

**Figure 2:**
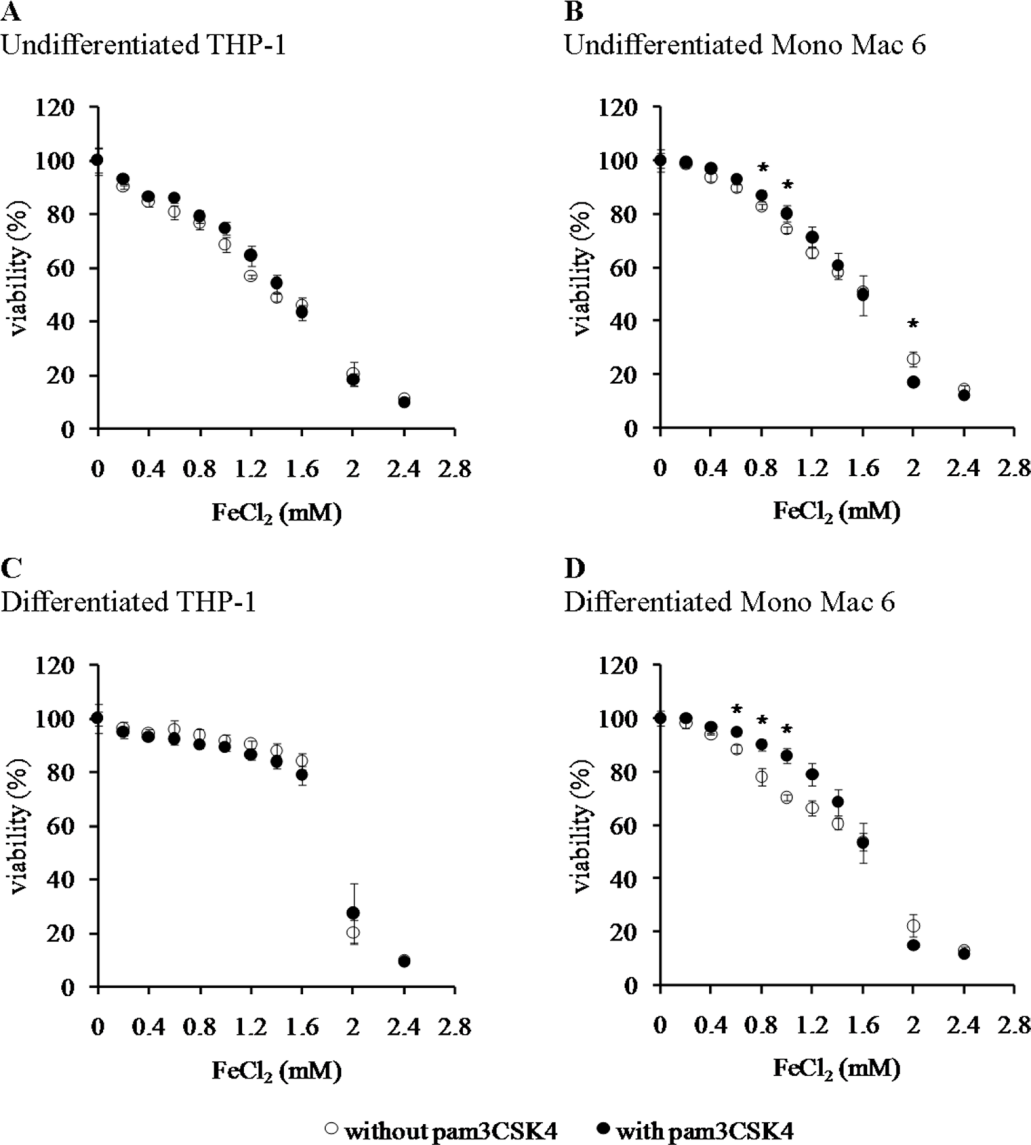
Viability of monocyte/macrophage cell lines upon exposure to a hydroxyl radical-generating system THP-1 (panels **A**, **C**) and Mono Mac 6 (panels **B**, **D**) cells were assayed without differentiation (panels A, B) or after differentiation as described in Materials and methods (panels C, D). Undifferentiated and differentiated cells were subsequently untreated (empty circles) or treated with 500 ng/ml pam3CSK4 (for TLR2 simulation) for 24 h (filled circles). Subsequently, cells were exposed to 1:10 (molar) mix of FeCl_2_ and ascorbic acid for 6 h and their viability was assayed with the resazurin method. Indicated concentrations refer to the concentration of FeCl_2_. Data presented as mean ± S.E.M. of percentage of viability in control (not exposed to oxidant) sample, *n* = 3. Asterisks indicate statistically significant difference between the TLR2-stimulated and unstimulated cells, *p <* 0.05.

**Figure 3:**
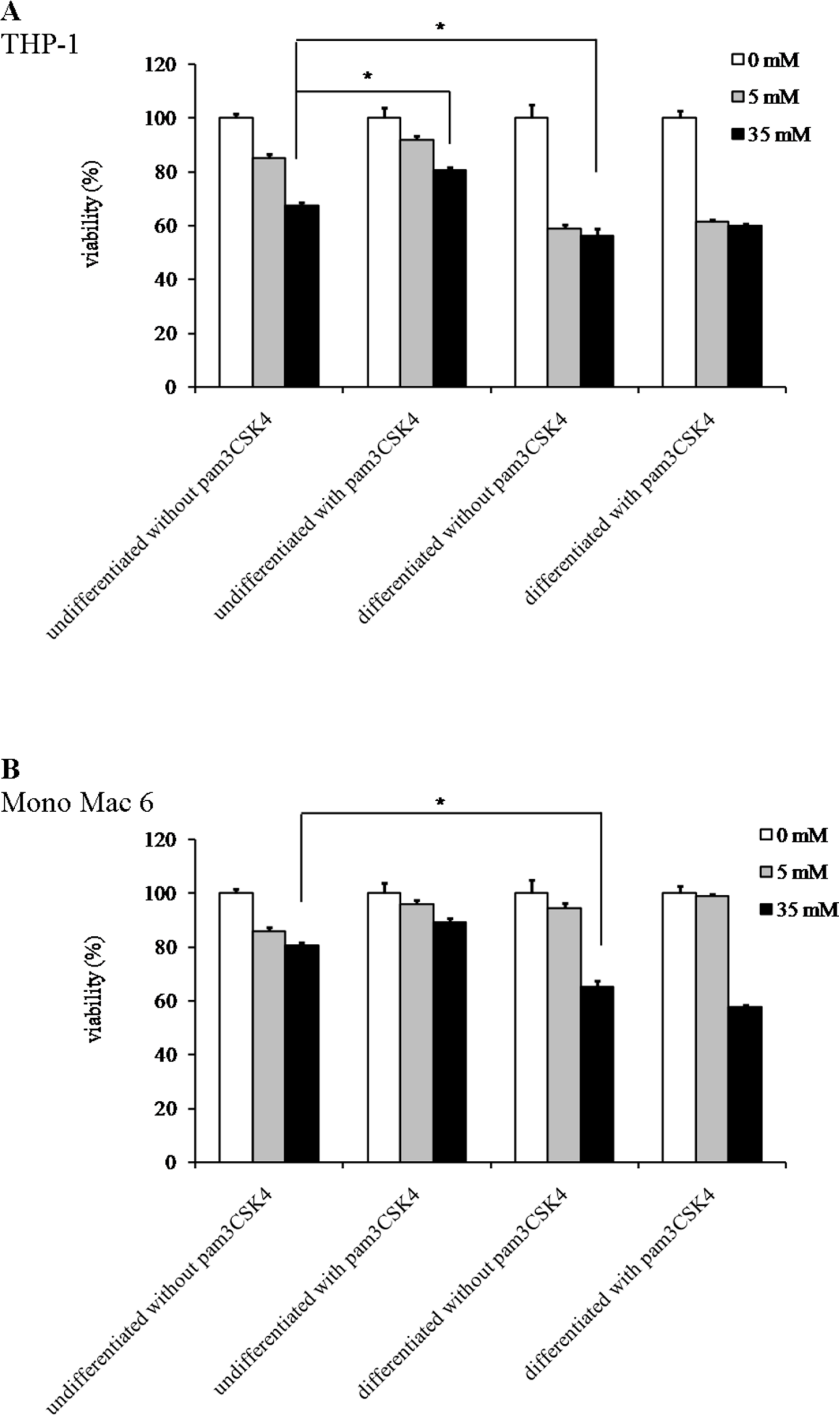
Viability of monocyte/macrophage cell lines upon exposure to paraquat THP-1 (panel **A**) and Mono Mac 6 (panel **B**) cells were assayed without differentiation or after differentiation as described in Materials and methods. Undifferentiated and differentiated cells were subsequently untreated or treated with 500 ng/ml pam3CSK4 (for TLR2 simulation) for 24 h. Subsequently, cells were exposed to indicated concentrations of paraquat and their viability was assayed with the resazurin method. Data presented as mean ± S.E.M. of percentage of viability in control (not exposed to oxidant) sample, *n* = 3. Asterisks indicate statistically significant difference between indicated samples, *p <* 0.05.

We found that the Mono Mac 6 cell line is several fold more resistant to hydrogen peroxide-derived oxidative stress than the THP-1 cell line, both in the undifferentiated state (IC50 is 1.12 ± 0.02 mM for THP-1 vs. 34.49 ± 1.88 mM for Mono Mac 6, Figure 1A, 1B) and differentiated state (IC50 is 1.84 ± 0.10 mM for THP-1 vs. 18.48 ± 1.11 mM for Mono Mac 6, Figure 1C, 1D). Interestingly, differentiation towards a more macrophage-like phenotype caused an increase in THP-1 resistance to H_2_O_2_ and a decrease in Mono Mac 6 resistance to the same challenge, although differentiated Mono Mac 6 cells still remain an order of magnitude more resistant than differentiated THP-1 cells. In contrast, resistance to the hydroxyl radical-generating reagents was similar in both cell lines in the undifferentiated state (IC50 is 1.39 ± 0.03 mM for THP-1 vs. 1.55 ± 0.02 mM for Mono Mac 6, Figure 2A, 2B), with THP-1 acquiring more resistance upon differentiation (IC50 is 1.81 ± 0.03 mM for THP-1 vs. 1.54 ± 0.03 mM for Mono Mac 6, Figure 2C, 2D). The pattern of resistance was different again for paraquat (superoxide) challenge—while Mono Mac 6 cells were slightly more resistant of two cell lines in the undifferentiated state, similar to resistance to hydrogen peroxide, differentiation actually caused a decrease in resistance to the same concentrations of the compound in both cell lines (Figure 3A and 3B), likely as a result of enhanced redox cycling due to higher availability of electron donors.

TLR2 activation had generally a (stronger or weaker) positive effect on cellular resistance to oxidative stress (Figures 1, 2, 3). Preincubation with the TLR2 agonist caused a highly significant increase in H_2_O_2_ resistance in both exposed cell lines (Figure 1): in THP-1 the increase in IC50 was more pronounced in the undifferentiated cells (from 1.12 ± 0.02 mM in non-activated cells to 1.50 ± 0.05 mM after pam3CSK4 activation) than in differentiated ones (from 1.84 ± 0.10 mM in non-activated cells to 1.97 ± 0.09 mM after pam3CSK4 activation), while in Mono Mac 6 the increase in IC50 was more pronounced in the differentiated state (from 18.48 ± 1.11 mM in non-activated cells to 32.48 ± 1.19 mM after pam3CSK4 activation) than in the undifferentiated state (from 34.49 ± 1.88 mM in non-activated cells to 36.56 ± 2.34 mM after pam3CSK4 activation). The impact of TLR2 activation on resistance to hydroxyl-generating mixture was minor and statistically significant only at few concentrations in the Mono Mac 6 cell line (Figure 2), with the only increase in IC50 (from 1.54 ± 0.03 mM in non-activated cells to 1.61 ± 0.02 mM after pam3CSK4 activation) noted in differentiated Mono Mac 6 cells. For the superoxide-generating compound, the increase of resistance was significant in the undifferentiated THP-1 cell line (Figure 3). Suppression of this TLR2 effect in differentiated cells may be linked to the same cause as the overall decline in paraquat resistance upon differentiation—increased electron availability overrides antioxidant defence effects.

### Activation of TLR2 increases the resistance of monocyte/macrophage cell lines to oxidative protein damage

**Figure 4:**
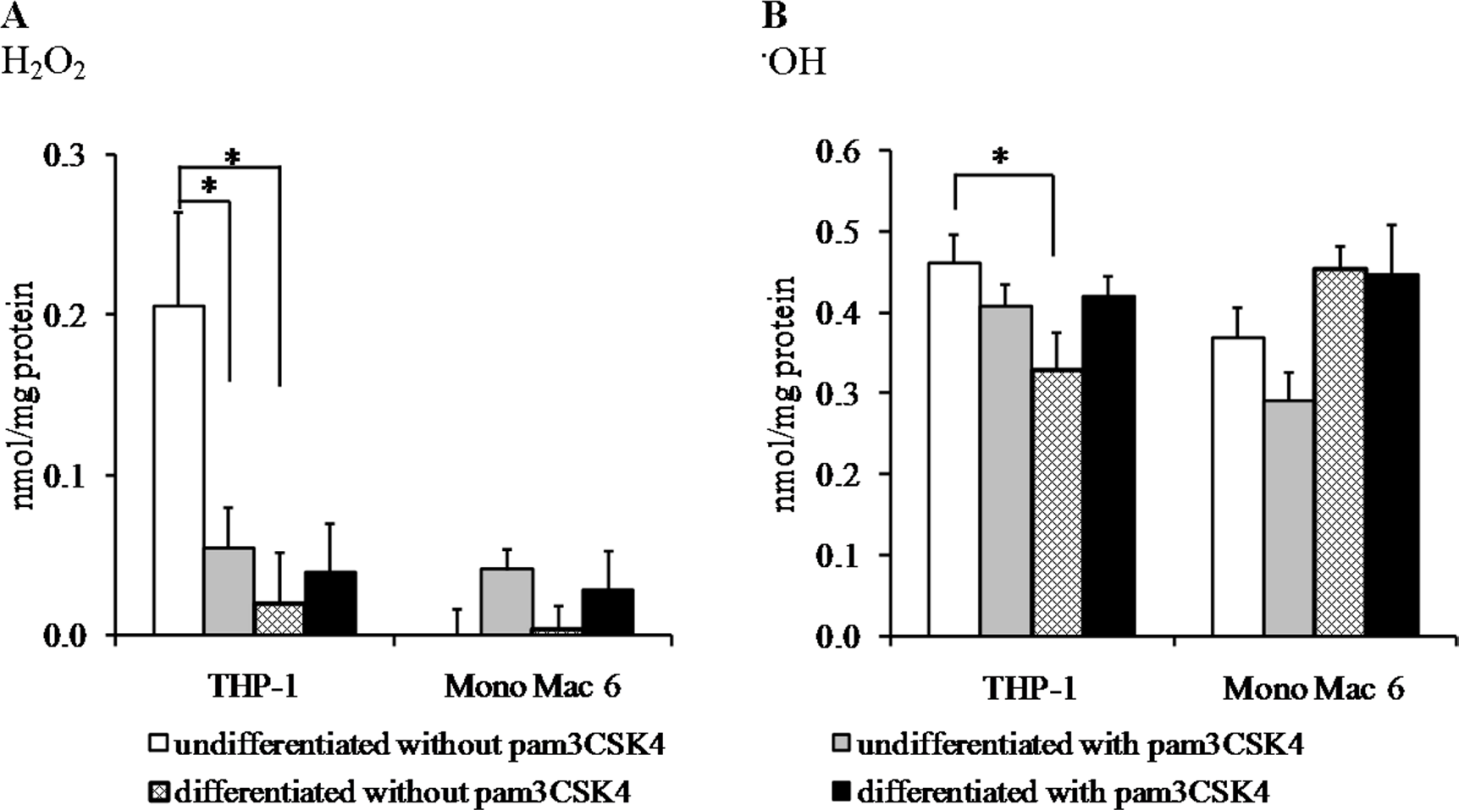
Oxidative damage in monocyte/macrophage cell lines upon exposure to reactive oxidants THP-1 and Mono Mac 6 cells were assayed without differentiation or after differentiation as described in Materials and methods. Undifferentiated and differentiated cells were subsequently untreated or treated with 500 ng/ml pam3CSK4 (for TLR2 simulation) for 24 h. Subsequently, cells were exposed to 1 mM hydrogen peroxide (panel **A**) or a hydroxyl radical-generating mix of 1 mM FeCl_2_ and 10 mM ascorbic acid (panel **B**) for 2 h and protein carbonyl group generation was assayed by the fluorescein-5-thiosemicarbazide method. Data presented as mean ± S.E.M. of nmol of carbonyl groups per mg of cellular protein, *n* = 6. Asterisks indicate statistically significant difference between indicated samples, *p <* 0.05.

We confirmed and supported our phenomenological survival data with measurements of actual oxidative damage effected upon cells under pro-oxidant challenge. We decided to measure protein oxidative damage in the form of free carbonyl groups as the most quantitative, easily measured hallmark which is directly relevant to health status of the cell regardless of its cell cycle status (oxidative damage to DNA is less likely to be important to survival of differentiated cells which stop dividing—see e.g. [32]). The most significant effect of macrophage differentiation on efficiency of antioxidant defence was recorded in the THP-1 cell line, where damage caused by H_2_O_2_ decreased over 10-fold after differentiation (Figure 4A). H_2_O_2_-caused damage was initially weaker in the Mono Mac 6 cell line (reflecting their above-mentioned higher survival upon H_2_O_2_ challenge), so that no damage at all was detectable either before or after differentiation. Due to these differences in H_2_O_2_-mediated damage in both cell lines, a significant decrease in damage marker level after TLR2 activation could be seen only in undifferentiated THP-1 cells (ca. 4-fold), while in Mono Mac 6 cells the damage actually increased (albeit non-significantly) after TLR2 activation. Ascorbate/Fe^2+^ caused very strong damage in both cell lines as expected, and neither differentiation nor TLR2 activation was able to decrease it significantly in most cases (a significant protective effect was seen only for differentiation in THP-1 cells), reflecting the instant reactivity of the hydroxyl radical and highlighting the potential role of antioxidant mechanisms downstream of the direct chemical damage to proteins in conferring increased resistance to this challenge (Figure 4B).

### Glutathione-related genes are involved in the antioxidant response of TLR2-stimulated cells

To identify molecular hallmarks of antioxidant defence associated with TLR2-mediated effects, we first embarked on a real-time PCR study of antioxidant gene expression. Based on copious literature data and our previous experience in antioxidant defence boosting by various stimuli, we divided the possible cellular mechanisms of antioxidant resistance into three generic systems: two thiol-related (glutathione system and thioredoxin system) and one enzymatic scavenger-related, reflecting reducing electron flow through glutathione, thioredoxin or directly through specialised antioxidant proteins/enzymes, respectively.

Differentiation caused a moderate-to-strong increase in the level of expression of several glutathione-related genes, more so in the THP-1 cell line than in the Mono Mac 6 cell line (Table 1A), suggesting a more pivotal role for the glutathione system at earlier stages of differentiation. The genes most conspicuously activated by differentiation in THP-1 cells were those encoding both subunits of the rate-limiting glutathione synthesis enzyme, γ-glutamylcysteine ligase (1.8 and 9.8-fold for the regulatory and catalytic subunit, respectively), several glutathione peroxidases (including peroxiredoxin 6, induced 2.4-fold) and glutaredoxin. This suggests an increased capability for GSH-mediated protein peroxidation removal, providing a mechanistic explanation for previous phenomenological results. In Mono Mac 6 cells, highest induction ratios are seen for glutathione peroxidase 7 (which is much more strongly expressed in these cells to begin with than in THP-1 cells) and for glutaredoxin.

**Table 1:** Antioxidant gene expression levels in monocyte/macrophage cell lines

A. Glutathione antioxidant system								
THP-1			Mono Mac 6					
Gene	Undifferentiated without pam3CSK4	Undifferentiated with pam3CSK4	Differentiated without pam3CSK4	Differentiated with pam3CSK4	Undifferentiated without pam3CSK4	Undifferentiated with pam3CSK4	Differentiated without pam3CSK4	Differentiated with pam3CSK4
GCLM	3728/318 (**100**)	11787^#^/789 (**316**)	6715*/989 (**180**)	11393/2769 (**306**)	4663/1118 (**100**)	10522^#^/567 (**226**)	3654/442 (**78**)	8825/2555 (**189**)
GCLC	522/36 (**100**)	763^#^/42 (**146**)	5108*/1209 (**979**)	5070/1075 (**972**)	948/144 (**100**)	1061/115 (**112**)	1782/810 (**188**)	2463/1235 (**260**)
GPX1	12121/2689 (**100**)	10521/1702 (**87**)	15846/3757 (**131**)	10645/1931 (**88**)	4980/202 (**100**)	4559/1 (**92**)	2996*/189 (**60**)	3517/81 (**71**)
GPX2	0.37/0.11 (**100**)	0.49/0.0076 (**131**)	0.59/0.021 (**157**)	1.09/0.13 (**292**)	0.74/0.0 (**100**)	0.81/0.26 (**109**)	0.82/0.30 (**110**)	1.93/0.024 (**260**)
GPX3	0.079/0.0 (**100**)	0.25/0.055 (**315**)	0.03*/0.0012 (**32**)	0.03^#^/0.0001 (**43**)	92/27 (**100**)	174/52 (**189**)	511/203 (**554**)	1999/675 (**2164**)
GPX4	12553/3123 (**100**)	12026/1003 (**96**)	31043*/3768 (**247**)	33628/5359 (**268**)	13939/2522 (**100**)	16841/291 (**121**)	10501/587 (**75**)	18180^#^/342 (**130**)
GPX7	306/12 (**100**)	198^#^/14 (**65**)	338/17 (**111**)	261^#^/4 (**85**)	2408/183 (**100**)	2336/251 (**97**)	8527/3013 (**354**)	6416/1857 (**266**)
GSS	891/147 (**100**)	860/7 (**97**)	1486/135 (**167**)	1205/107 (**135**)	1258/82 (**100**)	1329/142 (**106**)	1518/415 (**121**)	1870/528 (**149**)
GLRX	1093/176 (**100**)	454/46 (**42**)	1874*/23 (**171**)	2465/255 (**226**)	1835/188 (**100**)	1647/242 (**90**)	6027/2698 (**329**)	7356/3908 (**401**)
PRDX6	2578/126 (**100**)	3423^#^/221 (**133**)	6152*/970 (**239**)	6147/1055 (**238**)	3823/218 (**100**)	4490^#^/162 (**117**)	3764/254 (**98**)	3505/165 (**92**)
B. Thioredoxin antioxidant system								
TXN1	31184/2201 (**100**)	64624^#^/6876 (**207**)	47366*/5549 (**152**)	65865/11386 (**211**)	50048/8537 (**100**)	71227/4363 (**142**)	35069/1968 (**70**)	59814^#^/8123 (**120**)
TXN2	7691/805 (**100**)	6368/691 (**83**)	8708/415 (**113**)	6727/233 (**87**)	9187/299 (**100**)	10066/673 (**110**)	8871/1223 (**97**)	10557/1206 (**115**)
TXNRD1	261/26 (**100**)	1113^#^/74 (**427**)	1015*/93 (**389**)	1522^#^/109 (**583**)	295/48 (**100**)	570^#^/52 (**193**)	337/120 (**114**)	905/357 (**307**)
TXNRD2	48/36 (**100**)	12/19 (**24**)	37/10 (**76**)	84/57 (**173**)	131/8 (**100**)	130/14 (**99**)	174/55 (**133**)	196/64 (**149**)
PRDX1	33185/1935 (**100**)	68800^#^/3393 (**207**)	94574*/10818 (**285**)	115836/14433 (**349**)	85484/7345 (**100**)	115973/14342 (**136**)	69524/6176 (**81**)	98484/10697 (**115**)
PRDX3	11432/1970 (**100**)	8703/2313 (**76**)	18158/927 (**159**)	14364/2183 (**126**)	19118/1128 (**100**)	22056/1210 (**115**)	20573/1543 (**108**)	22342/340 (**117**)
SESN1	97/9 (**100**)	128/14 (**132**)	305*/20 (**314**)	194^#^/9 (**200**)	440/225 (**100**)	614/13 (**139**)	429/8 (**97**)	363/53 (**83**)
C. Direct scavenging enzymes								
SOD1	35940/2636 (**100**)	51887^#^/1365 (**144**)	55231*/3464 (**154**)	67622/6562 (**188**)	60076/4433 (**100**)	61554/4265 (**102**)	50342/3456 (**84**)	79362^#^/6110 (**132**)
SOD2	2573/256 (**100**)	164818^#^/6602 (**6406**)	45417*/6924 (**1765**)	322090^#^/42486 (**12518**)	12112/495 (**100**)	189804^#^/12061 (**1567**)	85927/49450 (**709**)	1404373^#^/479197 (**11595**)
CAT	1051/76 (**100**)	537^#^/49 (**51**)	579*/39 (**55**)	329^#^/29 (**31**)	3227/821 (**100**)	2896/361 (**90**)	3595/1296 (**111**)	1348/159 (**42**)

THP-1 and Mono Mac 6 cells were assayed without differentiation or after differentiation as described in Materials and Methods. Undifferentiated and differentiated cells were subsequently untreated or treated with 500 ng/ml pam3CSK4 (for TLR2 simulation) for 6 h. Subsequently, gene expression was assayed at the mRNA level by the real-time RT-PCR method for genes corresponding to the glutathione antioxidant system (Table 1A), thioredoxin antioxidant system (Table 1B) and direct scavenging enzymes (Table 1C). Data expressed as relative mRNA copy number per 1000 copies of averaged reference mRNA, calculated by 2^-∆Ct^ transformation, presented as mean / transformed upper S.E.M. and separately (below, in parenthesis) as percentage of expression level in the undifferentiated/unstimulated sample; *n* = 3. Asterisks indicate statistically significant difference between the undifferentiated and differentiated cells, *p <* 0.05; hash signs indicate statistically significant difference between the TLR2-stimulated and unstimulated cells, *p <* 0.05.

The effect of TLR2 activation on glutathione system gene expression was comparable to that of macrophage-like differentiation in undifferentiated monocyte-like cells, and in some cases was notable even in already differentiated cells where induction of gene expression from the basal level had already taken place (Table 1A). Most notably, TLR2 activation caused a strong (1.7 to 3.2-fold) increase in *GCLM* (regulatory subunit of γ-glutamylcysteine ligase) expression in all cellular backgrounds. Also peroxiredoxin 6 and *GPX3* (two of glutathione-dependent peroxidases) were induced in undifferentiated monocyte-like cells. We verified the involvement of TLR2 in the investigated effect by inhibiting the receptor with a specific blocking antibody. The observed inhibition of pam3CSK4-induced stimulation of glutathione system gene expression was consistent with TLR2 participation, e.g. GCLM induction in undifferentiated THP-1 cells decreased by 25%.

### The glutathione antioxidant system is involved in the antioxidant response of TLR2-stimulated cells

**Figure 5:**
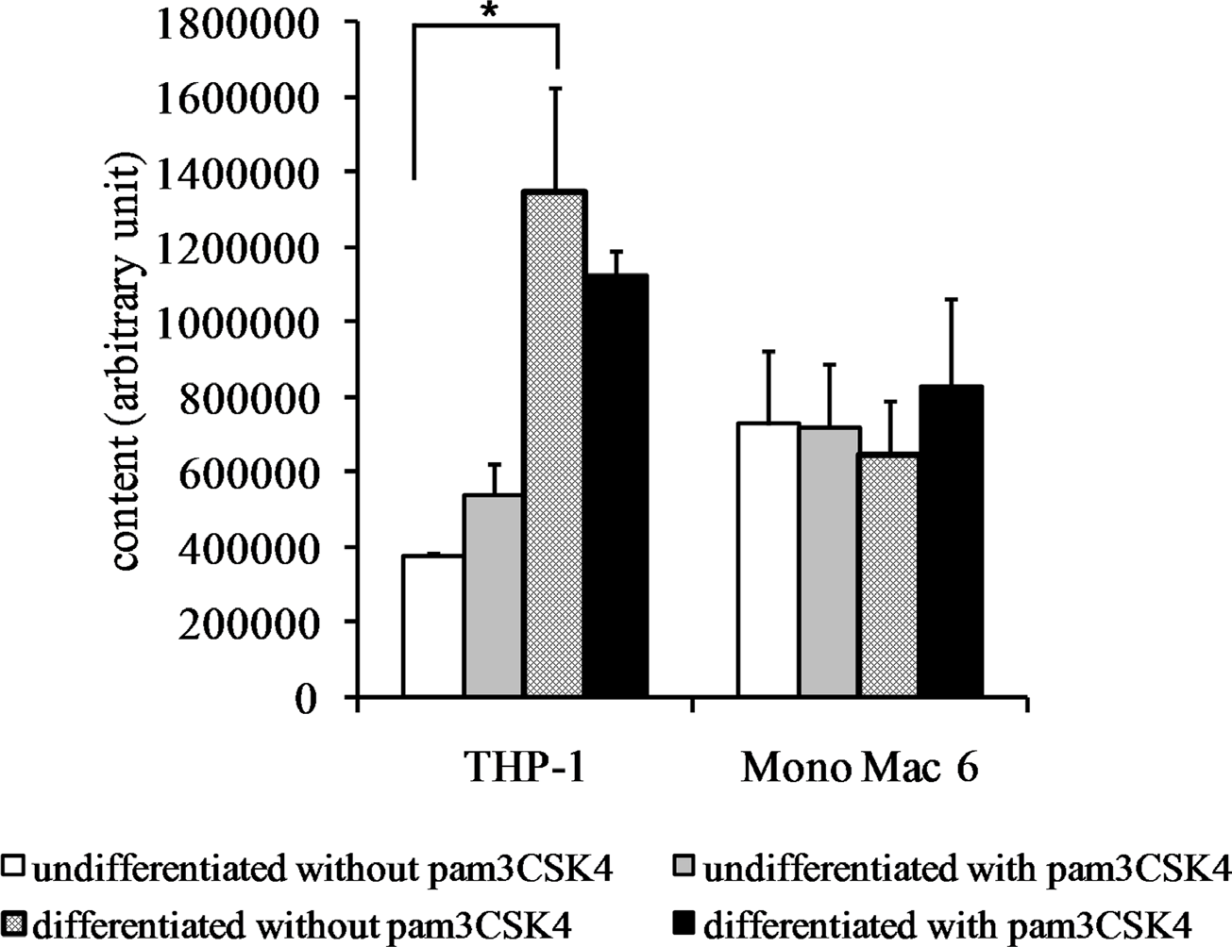
Glutathione content in monocyte/macrophage cell lines THP-1 and Mono Mac 6 cells were assayed without differentiation or after differentiation as described in Materials and methods. Undifferentiated and differentiated cells were subsequently untreated or treated with 500 ng/ml pam3CSK4 (for TLR2 simulation) for 24 h. Subsequently, glutathione content was assayed by the o-phthalaldehyde method. Data presented as mean ± S.E.M. of glutathione-phthalaldehyde adduct fluorescence, *n* = 3. Asterisks indicate statistically significant difference between indicated samples, *p <* 0.05.

Confirming the above mRNA-level data, we detected a very significant (over 3-fold) increase in steady-state reduced glutathione levels in the THP-1 cell line upon differentiation (Figure 5). The 1.4-fold increase in the same cell type upon TLR2 agonist challenge was not statistically significant; also, the glutathione level in differentiated THP-1 cells could not be further increased by TLR2 activation as it was already very high. This was also the case for both undifferentiated and differentiated Mono Mac 6 cells, for which their GSH level could not be significantly further augmented by any used treatment (their basal GSH level was higher than in THP-1 cells).

**Figure 6:**
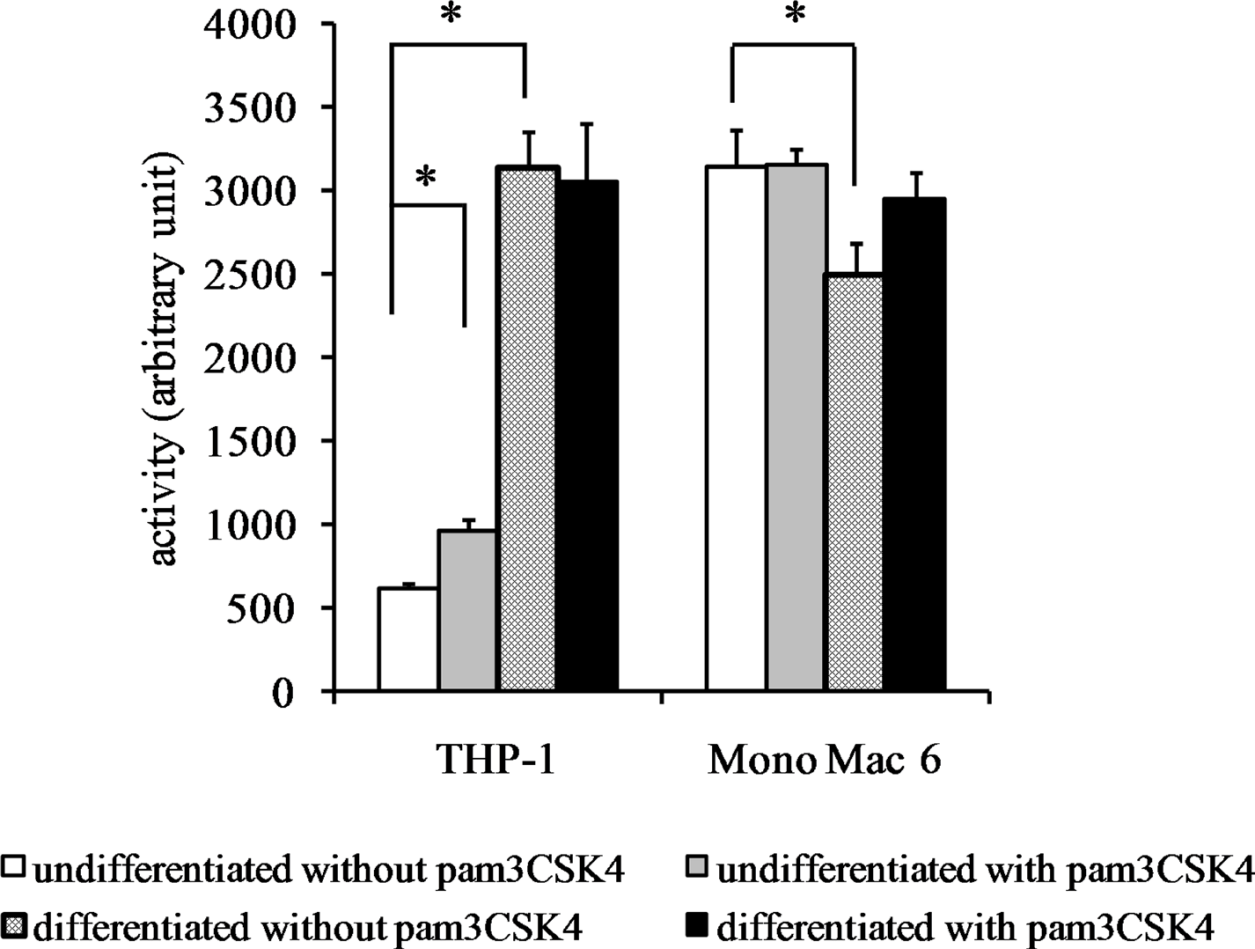
Glutathione reductase activity in monocyte/macrophage cell lines THP-1 and Mono Mac 6 cells were assayed without differentiation or after differentiation as described in Materials and methods. Undifferentiated and differentiated cells were subsequently untreated or treated with 500 ng/ml pam3CSK4 (for TLR2 simulation) for 24 h. Subsequently, glutathione reductase was assayed by the NADPH consumption method. Data presented as mean ± S.E.M. in arbitrary NADPH consumption units, *n* = 3. Asterisks indicate statistically significant difference between indicated samples, *p <* 0.05.

**Figure 7:**
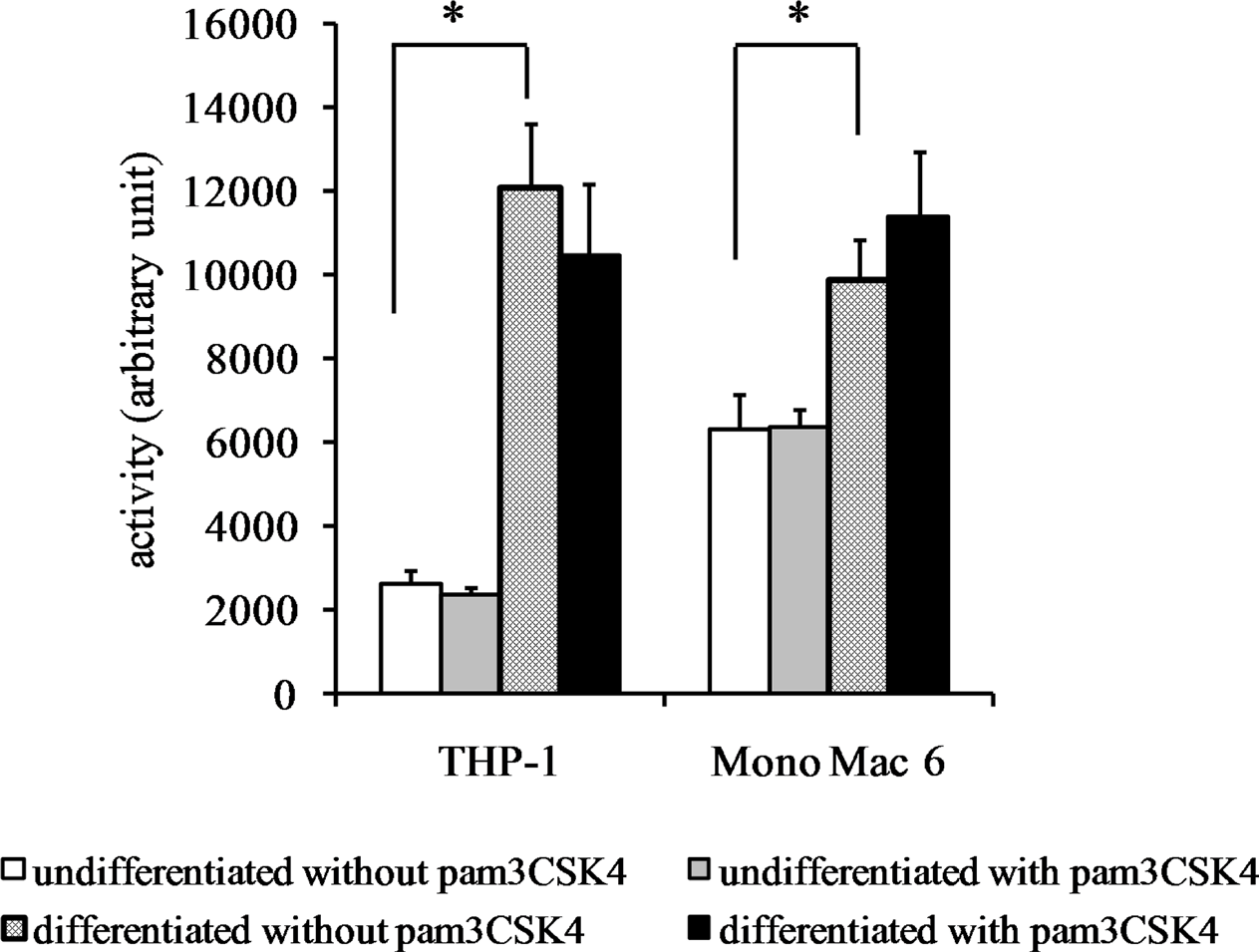
Glutathione peroxidase activity in monocyte/macrophage cell lines THP-1 and Mono Mac 6 cells were assayed without differentiation or after differentiation as described in Materials and methods. Undifferentiated and differentiated cells were subsequently untreated or treated with 500 ng/ml pam3CSK4 (for TLR2 simulation) for 24 h. Subsequently, glutathione peroxidase was assayed by the glutathione reductase coupling/NADPH consumption method. Data presented as mean ± S.E.M. in arbitrary NADPH consumption units, *n* = 3. Asterisks indicate statistically significant difference between indicated samples, *p <* 0.05.

We also verified the total cellular activity of glutathione reductase and glutathione peroxidase-like enzymes. For glutathione reductase activity (Figure 6), a significant increase could be seen upon differentiation of THP-1 cells (4.6-fold) and (weaker, 1.4-fold) upon TLR2 agonist challenge of undifferentiated THP-1 cells. No significant increases in GR activity were seen in Mono Mac 6 cells. For total glutathione peroxidase (encompassing several individual enzymatic proteins), differentiation caused an increase of enzymatic activity both in THP-1 and Mono Mac 6 cell lines (3-fold and 1.4-fold, respectively), and TLR2 activation was ineffective in changing it further (Figure 7).

The overall contribution of the glutathione system to antioxidant defence was verified by performing viability assay after hydrogen peroxide challenge (at a single near-IC50 concentration of 1.5 mM for THP-1 cell and 40 mM for Mono Mac 6 cells) for cells preincubated with buthionine sulfoximine (inhibitor of GSH synthesis) at 0.5 mM and carmustine (inhibitor of glutathione reductase) at 0.1 mM. BSO was effective in increasing H_2_O_2_ toxicity in THP-1 cells (from 32.8% ± 2.3% to 22.5% ± 0.6% viability for undifferentiated cells, from 35.8% ± 2.8% to 22.3% ± 2.4% viability for differentiated cells), while carmustine had no effect (data not shown). In the Mono Mac 6 line, BSO increased H_2_O_2_ toxicity only in differentiated cells (from 42.6% ± 0.2% to 31.0% ± 3.6% viability) and carmustine did not increase it (data not shown). BSO pre-treatment increased H_2_O_2_ toxicity in THP-1 cells treated with pam3CSK4 (from 51.2% ± 7.5% to 30.8% ± 7.2% viability for undifferentiated cells, from 28.6% ± 3.5% to 16.5% ± 2.6% viability for differentiated cells).

### Thioredoxin-related genes are involved in the antioxidant response of TLR2-stimulated cells

Within the thioredoxin system, commonly considered a fine-tuning element of cellular antioxidant resistance, we detected few significant effects of differentiation of monocyte-like cells on gene expression at mRNA level (Table 1B). No thioredoxin-related genes were significantly induced by differentiation of Mono Mac 6 cells, and differentiating THP-1 cells only caused notable increases in expression of genes encoding thioredoxin reductase-1 (3.9-fold) and sestrin-1 (3.1-fold). On the other hand, treating cells with the TLR2 agonist activated more thioredoxin-related genes, both in THP-1 and in Mono Mac 6 cells, usually independent on their stage of differentiation. The mRNA level for cytoplasmic thioredoxin itself (product of the *TXN1* gene) increased in all TLR2 agonist-treated cell types by a factor between 1.4 and 2.1. Also the expression levels of most abundant isoenzymes of thioredoxin reductase and thioredoxin peroxidase (peroxiredoxin) were uniformly induced in investigated cells, with a synergistic effect of differentiation and TLR2 stimulation: induction ratio towards undifferentiated and unchallenged cells for *TXNRD1* was as high as 5.8 for THP-1 and 3.1 for Mono Mac 6. We verified the involvement of TLR2 in the investigated effect by inhibiting the receptor with a specific blocking antibody. The observed inhibition of pam3CSK4-induced stimulation of thioredoxin system gene expression was consistent with TLR2 participation, e.g. TXN1 induction in undifferentiated THP-1 cells decreased by 22%.

### The thioredoxin antioxidant system is involved in the antioxidant response of TLR2-stimulated cells

**Figure 8:**
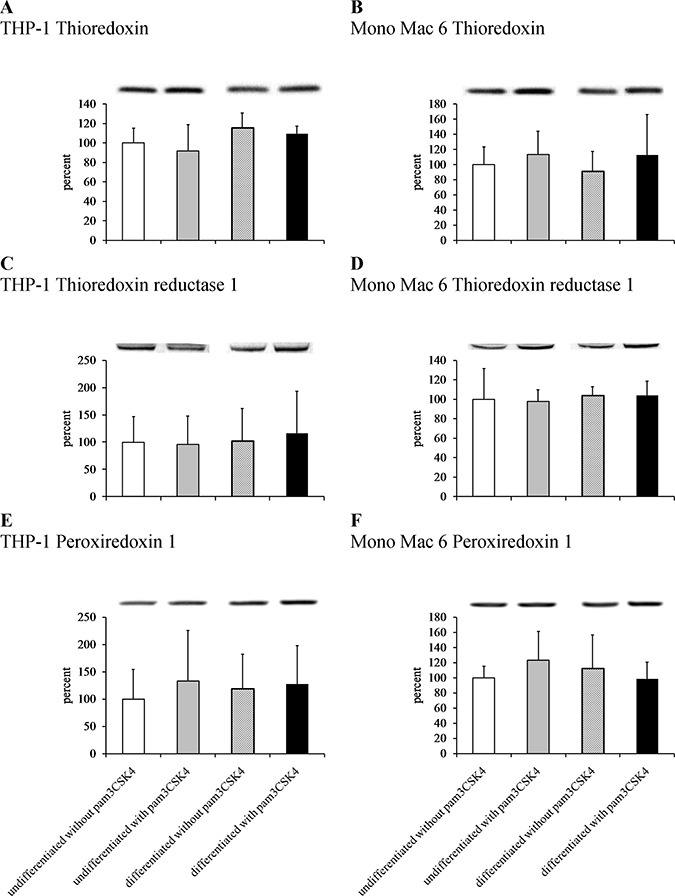
Thioredoxin antioxidant system protein content in monocyte/macrophage cell lines THP-1 (panels **A**, **C**, **E**) and Mono Mac 6 (panels **B**, **D**, **F**) cells were assayed without differentiation or after differentiation as described in Materials and methods. Undifferentiated and differentiated cells were subsequently untreated or treated with 500 ng/ml pam3CSK4 (for TLR2 simulation) for 24 h. Subsequently, protein content was assayed by Western blotting with antibodies against thioredoxin (panels A, B), thioredoxin reductase 1 (panels C, D) and peroxiredoxin 1 (panels E, F). Data presented as mean ± S.E.M. of ratio of Western blot band intensity corresponding to the assayed protein to Western blot band intensity corresponding to β-actin, assayed in the same sample, expressed as percentage of value for the undifferentiated/unstimulated sample; *n* = 3. Representative blot images are presented above respective data points.

**Figure 9:**
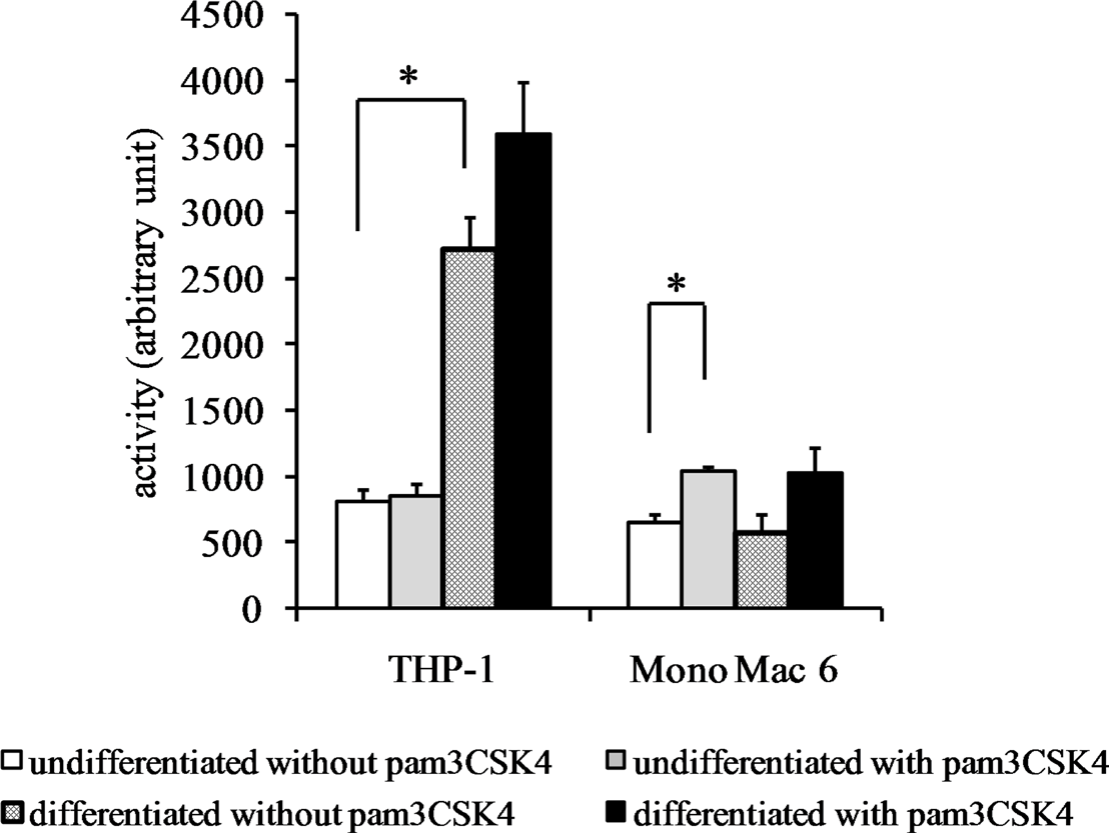
Thioredoxin reductase activity in monocyte/macrophage cell lines THP-1 and Mono Mac 6 cells were assayed without differentiation or after differentiation as described in Materials and methods. Undifferentiated and differentiated cells were subsequently untreated or treated with 500 ng/ml pam3CSK4 (for TLR2 simulation) for 24 h. Subsequently, thioredoxin reductase was assayed by the DTNB method. Data presented as mean ± S.E.M. in arbitrary TNB production units, *n* = 3. Asterisks indicate statistically significant difference between indicated samples, *p <* 0.05.

We sought to confirm these results at protein level by immunoblotting cell extracts, but we were unable to obtain reproducible and statistically significant increases in respective protein levels (Figure 8). However, an enzymatic activity assay for thioredoxin reductase activity (Figure 9) led to results similar to those derived from gene expression assays, namely an induction of thioredoxin reductase activity upon differentiation of THP-1 cells (3.3-fold) and upon TLR2 activation (1.3-fold in differentiated THP-1 cells, 1.6-fold in undifferentiated Mono Mac 6 cells and 1.8-fold in differentiated Mono Mac 6 cells). This effect may be related to the cited fine-tuning character of the thioredoxin system at large, where relatively slight changes of protein expression level and/or protein localisation may exert unproportionally large impact upon cellular redox homeostasis.

Analogously to the glutathione system, we verified the contribution of the thioredoxin system to antioxidant defence by performing viability assay after hydrogen peroxide challenge for cells preincubated with auranofin (inhibitor of thioredoxin reductase) at 0.5 μM. It did not increase H_2_O_2_ toxicity in the two cell lines under study, either undifferentiated or differentiated (data not shown).

### ROS-scavenging antioxidant enzymes are involved in the antioxidant response of TLR2-stimulated cells

With regard to main direct ROS-scavenging cellular enzymes, we were surprised to see no induction of catalase expression at all, with actually statistically significant decrease in catalase mRNA expression in THP-1 cells both upon differentiation and upon TLR2 activation (Table 1C). This lack of catalase involvement in observed induction of antioxidant defence could be confirmed by enzymatic activity assay (data not shown). On the other hand, superoxide dismutases, especially the manganese SOD (encoded by the *SOD2* gene), underwent dramatic induction. Strongly synergistic effects of differentiation and TLR2 activation caused *SOD2* mRNA expression to rise from values lower than those for *SOD1* to becoming the dominant SOD gene expressed. The induction ratio of SOD2 by TLR2 agonist exposure varied between 15.7 for undifferentiated Mono Mac 6 cells and 64.1 for undifferentiated THP-1 cells (Table 1C). We verified the involvement of TLR2 in the investigated effect by inhibiting the receptor with a specific blocking antibody. The observed inhibition of pam3CSK4-induced stimulation of antioxidant enzyme gene expression was consistent with TLR2 participation, e.g. *SOD2* induction in undifferentiated THP-1 cells decreased by 24%.

**Figure 10:**
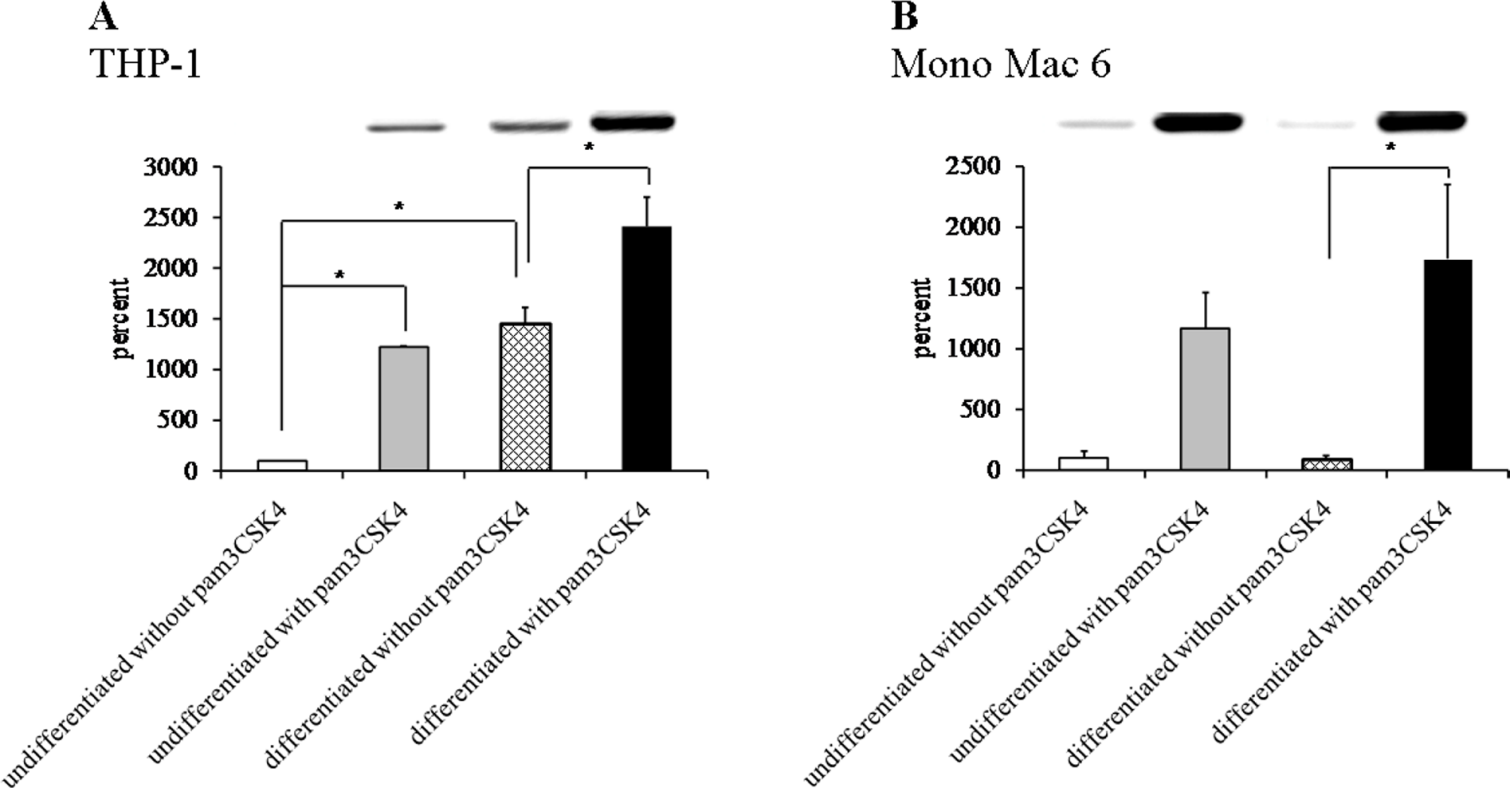
Superoxide dismutase protein content in monocyte/macrophage cell lines THP-1 (panel **A**) and Mono Mac 6 (panel **B**) cells were assayed without differentiation or after differentiation as described in Materials and methods. Undifferentiated and differentiated cells were subsequently untreated or treated with 500 ng/ml pam3CSK4 (for TLR2 simulation) for 24 h. Subsequently, protein content was assayed by Western blotting with antibodies against superoxide dismutase 2. Data presented as mean ± S.E.M. of ratio of Western blot band intensity corresponding to the assayed protein to Western blot band intensity corresponding to β-actin, assayed in the same sample, expressed as percentage of value for the undifferentiated/unstimulated sample; *n* = 3. Representative blot images are presented above respective data points. Asterisks indicate statistically significant difference between indicated samples, *p <* 0.05.

**Figure 11:**
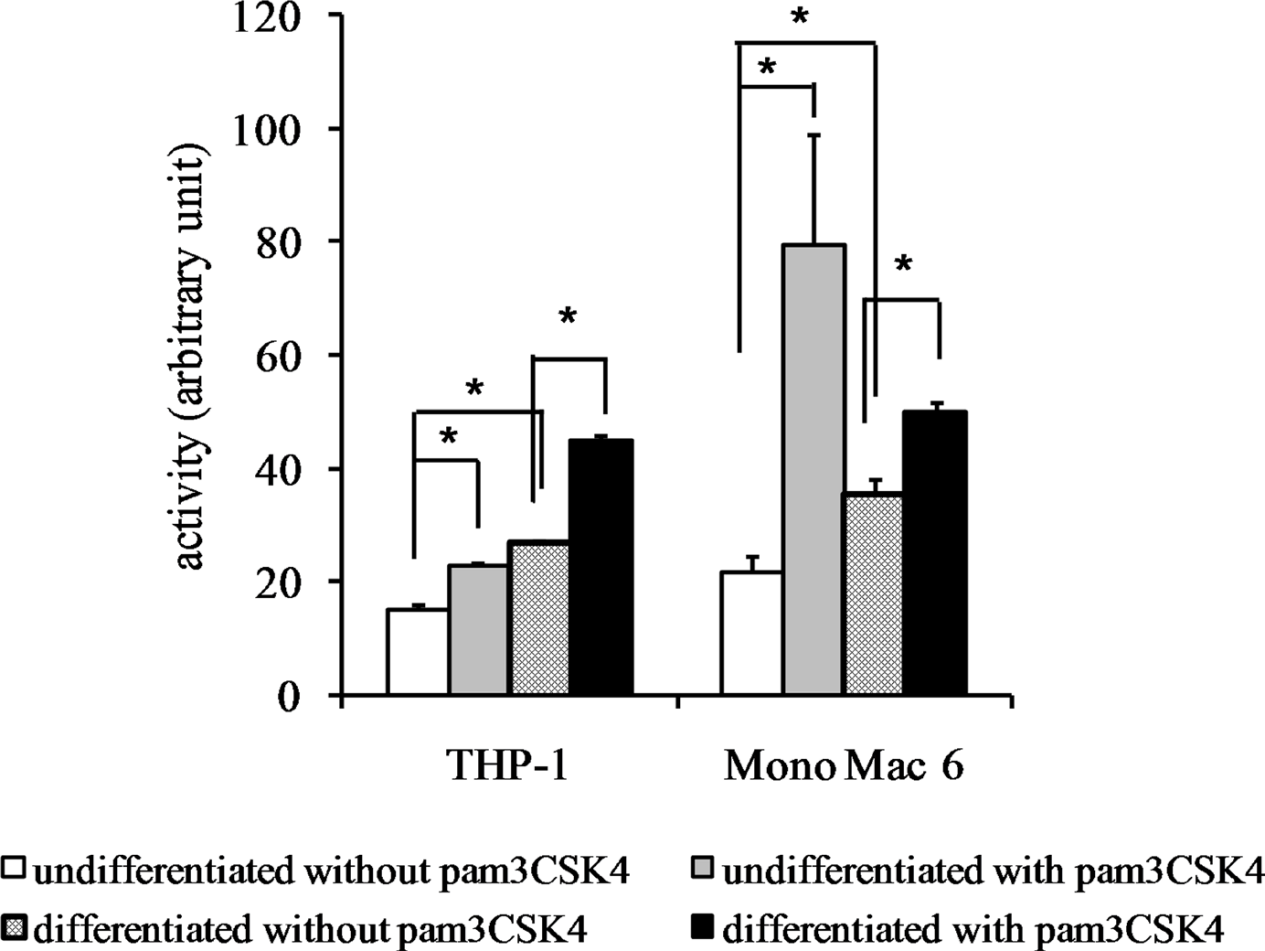
Superoxide dismutase activity in monocyte/macrophage cell lines THP-1 and Mono Mac 6 cells were assayed without differentiation or after differentiation as described in Materials and methods. Undifferentiated and differentiated cells were subsequently untreated or treated with 500 ng/ml pam3CSK4 (for TLR2 simulation) for 24 h. Subsequently, superoxide dismutase was assayed by the nitro blue tetrazolium method. Data presented as mean ± S.E.M. in arbitrary blue formazan production units, *n* = 3. Asterisks indicate statistically significant difference between indicated samples, *p <* 0.05.

The impressive superoxide dismutase induction was corroborated by protein- and activity-level data. We were able to confirm robust SOD2 induction at the protein level (Figure 10) by differentiation (14.6-fold) and by TLR2 activation (12.2-fold) in THP-1 cells and by TLR2 activation in Mono Mac 6 cells (11.7-fold, rising to 17.4-fold in differentiated cells). Dismutase enzymatic activity was also induced (Figure 11) by both differentiation and TLR2 agonist treatment, with induction ratios ranging from 1.5 for THP-1 cells to 3.7 for Mono Mac 6 cells. A strong synergy of differentiation and TLR2 action is noticeable especially for THP-1 cells.

Chemical inhibitors of direct antioxidant enzymes were used to verify their importance for antioxidant defence, in analogy to experiments with the thiol systems. We performed a viability assay after hydrogen peroxide exposure at near-IC50 values, for cells preincubated with diethyldithiocarbamate (DDC, SOD inhibitor) at 0.5 mM and sodium azide (catalase inhibitor) at 1 μM. Interestingly, while DDC pre-treatment increased H_2_O_2_ toxicity only for THP-1 cells (from 32.8% ± 2.3% to 16.2% ± 1.1% viability for undifferentiated cells, from 35.8% ± 2.8% to 27.0% ± 2.5% viability for differentiated cells) and not for Mono Mac 6 (data not shown), sodium azide was effective both in THP-1 cells (from 32.8% ± 2.3% to 22.6% ± 1.6% viability for undifferentiated cells, from 35.8% ± 3.5% to 22.6% ± 2.0% viability for differentiated cells) and in differentiated Mono Mac 6 cells (from 42.6% ± 0.2% to 32.7% ± 2.1% viability). DDC pre-treatment increased H_2_O_2_ toxicity for THP-1 cells treated with pam3CSK4 (from 51.2% ± 7.5% to 22.2% ± 5.5% viability for undifferentiated cells, from 28.6% ± 3.5% to 21.7% ± 2.9% viability for differentiated cells).

### ROS-insensitive calcium channels are induced by macrophage differentiation and TLR2 stimulation

**Figure 12:**
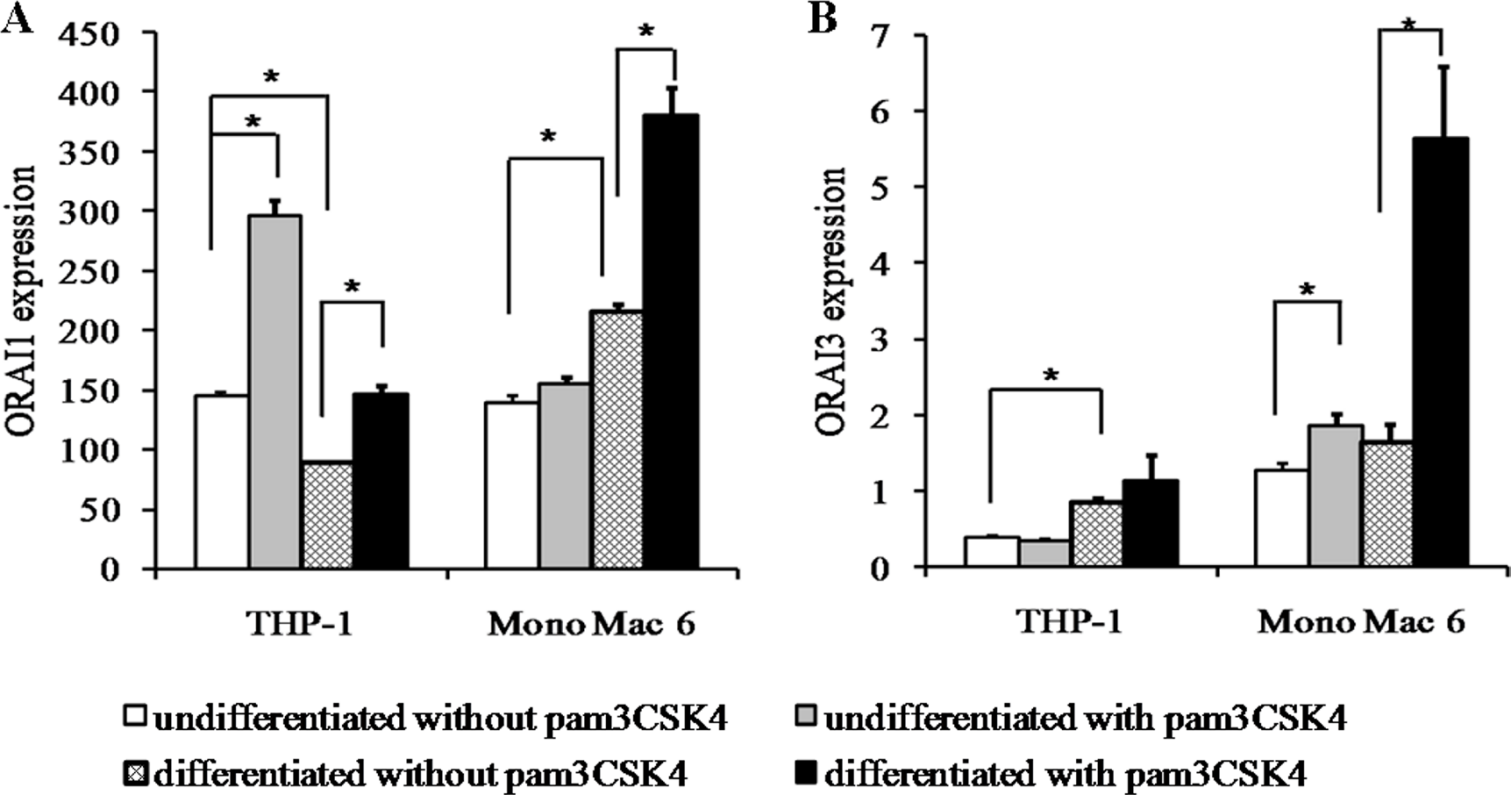
ORAI calcium channel isoform expression in monocyte/macrophage cell lines THP-1 and Mono Mac 6 cells were assayed without differentiation or after differentiation as described in Materials and methods. Undifferentiated and differentiated cells were subsequently untreated or treated with 500 ng/ml pam3CSK4 (for TLR2 simulation) for 6 h. Subsequently, *ORAI1* (**A**) and *ORAI3* (**B**) gene expression was assayed at the mRNA level by the real-time RT-PCR method. Data expressed as relative mRNA copy number per 1000 copies of averaged reference mRNA, calculated by 2^-∆Ct^ transformation, presented as mean ± transformed upper S.E.M.; *n* = 3. Asterisks indicate statistically significant difference between indicated samples, *p <* 0.05.

Since the relative abundance of ROS-sensitive (ORAI1) and -insensitive (ORAI3) calcium channels involved in store-operated calcium entry has recently been implicated both in redox regulation of monocyte/macrophage function and in oxidative stress resistance in these cell types [33], we verified the expression levels of genes coding for these channels in our cellular models. While the ROS-sensitive isoform (*ORAI1*) showed induction or moderate repression ratios upon differentiation and TLR2 stimulation in both cell lines (Figure 12), the ROS-insensitive *ORAI3* isoform demonstrated consistent induction by TLR2 ligand, which in the case of Mono Mac 6 cells was strongly synergistic with induction by differentiation (pam3CSK4 induces the expression 1.46-fold, differentiation, 1.28-fold, pam3CSK4 in differentiated cells, 4.43-fold). Concomitantly, the *ORAI3*/*ORAI1* ratio, suggested previously to be a marker of the calcium entry—redox homeostasis feedback loop in monocytes, increased significantly in TLR2-stimulated Mono Mac 6 cells (undifferentiated, from 9.2 to 12.0; differentiated, from 7.6 to 14.8), but not in TLR2-stimulated THP-1 cells, where *ORAI1* induction exceeded increase in *ORAI3* expression.

### Nrf2 expression and subcellular localisation is modulated by TLR2 stimulation in THP-1 cells

**Figure 13:**
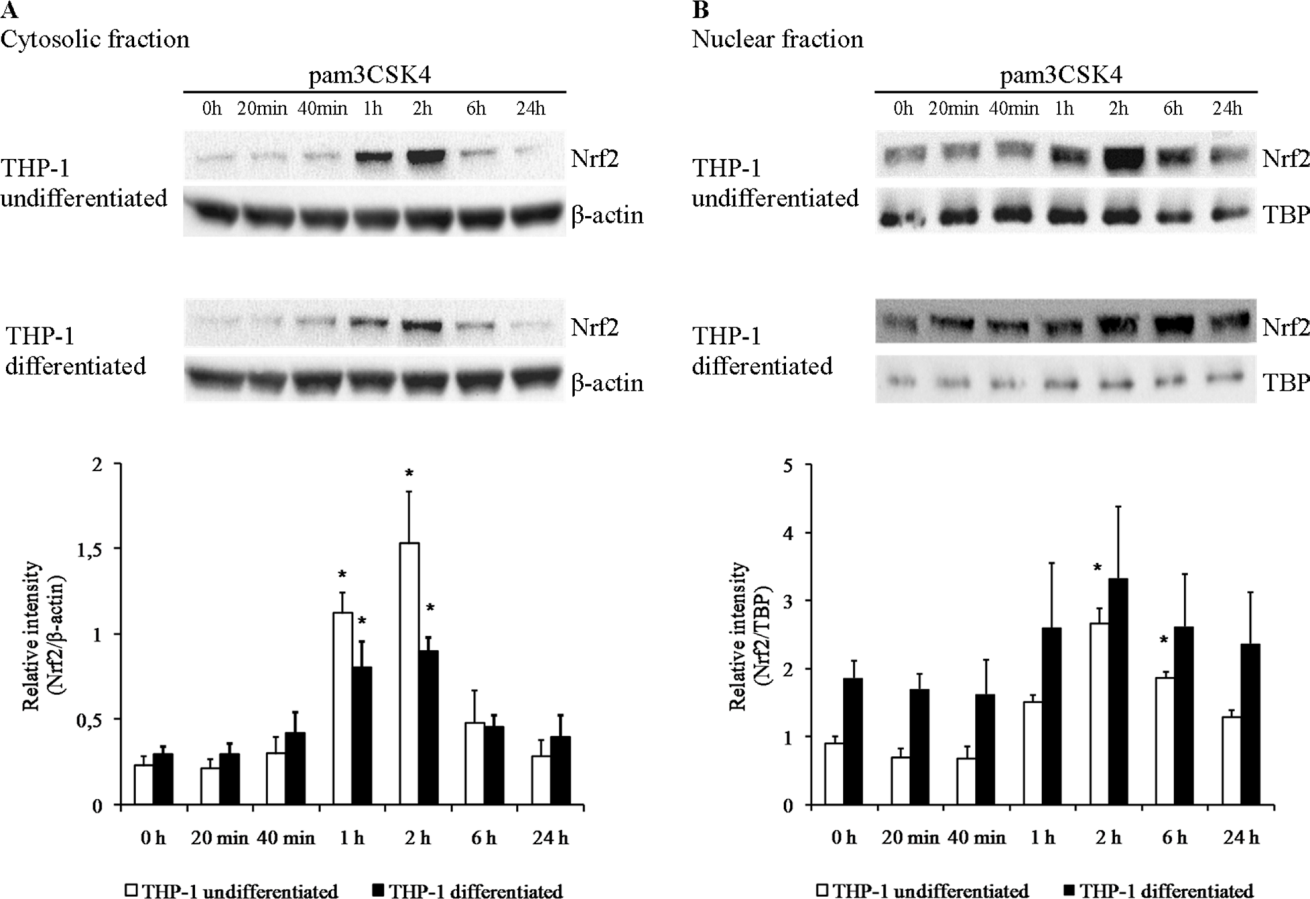
Nrf2 protein levels are increased by TLR2 activation in THP-1 cells THP-1 cells were assayed without differentiation or after differentiation as described in Materials and methods. Undifferentiated and differentiated cells were subsequently untreated or treated with 500 ng/ml pam3CSK4 (for TLR2 simulation) for indicated periods of time. Subsequently, protein content was assayed by Western blotting with antibodies against Nrf2 in cytoplasmic (**A**) and nuclear (**B**) fractions isolated as described in Materials and methods. Data presented as representative blot images and graphs of mean ± S.E.M. of ratio of Western blot band intensity corresponding to the assayed protein to Western blot band intensity corresponding to β-actin (A) or TBP (B), assayed in the same sample; *n* = 3. Asterisks indicate statistically significant difference between sample and the non-stimulated control, *p <* 0.05.

Since many antioxidant genes studied here are under common regulation by the redox-sensitive transcription factor Nrf2, we decided to verify whether its concentration in separate cellular compartments (cytoplasm, where it remains latent, and nucleus, where it is transcriptionally active) changes in cellular models of macrophage differentiation and TLR2 activation. While overall expression level of Nrf2 in the Mono Mac 6 cell line was too low for reliable measurement (data not shown), Western blotting of extracts from THP-1 cells revealed that PMA differentiation of this cell line leads to a ca. two-fold increase of the amount of nuclear Nrf2 with no significant change of cytoplasmic levels (Figure 13). Moreover, there was a striking and transient increase of both cytoplasmic and nuclear Nrf2 level in this cell line upon pam3CSK4 stimulation, which peaked at ca. 2 h from stimulation and subsequently receded. The amplitude of this increase was larger in undifferentiated THP-1 cells (ca. 6.7-fold in the cytoplasm and 3.0-fold in the nucleus) than in differentiated ones (ca. 3.1-fold in the cytoplasm and 1.8-fold in the nucleus).

## DISCUSSION

It is known that macrophages are subject to strong oxidative challenges during their function in tissues, both from exogenous and endogenous sources [9–11]. Macrophages are also reported to have a correspondingly strong antioxidant defence [13–14].

It is not clear a) whether this defence arises early in differentiation and is present in significantly increased amounts already in circulating monocytes or it appears upon terminal differentiation, along with macrophage markers; b) what are the molecular mechanisms of this defence, which compounds and enzymes take part; c) whether this defence can be further modulated by stimuli that activate/regulate macrophage function, such as PRR ligands.

In our study, we have provided data and at least partial answers to these three questions, using *in vitro* cell line models of monocyte-macrophage differentiation. While such models are necessarily not perfect, having additional homeostatic changes stemming from both immortalisation and adaptation to *in vitro* culture conditions, they are commonly used at the identification/verification stage of biochemical hypotheses. They usually retain basic transcriptional and biochemical molecular mechanisms in comparison to their cellular counterparts *in vivo* [7, 8].

The overall conclusion from the first stage of experiments, related to phenomenological observations on oxidative stress resistance, points to early differentiation stages as the period of appearance of increased resistance. The phenotypically less mature cell line THP-1 [7] is relatively weakly resistant to hydrogen peroxide and this resistance increases significantly upon differentiation; Mono Mac 6, which has more in common with mature macrophages [8], has also a significantly higher resistance level which does not increase further upon differentiation (which is accepted as a model of terminal macrophage differentiation). This higher capacity for increase in resistance upon differentiation in THP-1 is also seen for a more aggressive oxidative challenge by hydroxyl radical generation, confirming the general character of resistance enhancement at early differentiation stages. Moreover, the relevance of cellular redox homeostasis for detected differences in cell survival is confirmed by a substantial decrease of peroxide-mediated and hydroxyl radical-mediated oxidative damage to proteins upon THP-1 differentiation. This line of evidence is consistent with some previous studies [34, 35]. It is important to remember that the two cell lines we have studied are genetically and phenotypically distinct and that Mono Mac 6 should not be interpreted simplistically as a further differentiation stage relative to the THP-1 phenotype. However, the indubitably closer phenotypical relation of Mono Mac 6 cells to macrophages, together with parallel comparison of results between undifferentiated and differentiated forms of each cell line, provides a clear message on the positive relation between direction of the differentiation process and strengthening of antioxidant defence. It is interesting to note that it was more difficult for us to pharmacologically decrease oxidative stress resistance (by the use of enzyme-specific inhibitors) in the Mono Mac 6 cell line than in the THP-1 cell line, suggesting that resistance mechanisms in more terminally differentiated macrophage-type cells are more redundant and complex, with overlapping competence.

Since defence from artificial oxidative stress is a complicated process involving not only cellular antioxidants, but also e.g. suppression of reactive oxidant generation or their efficient compartmentalisation, phenomenological effects of differentiation were not equal in all experimental setups used in the present study. Resistance to the redox-cycling superoxide generator, paraquat, was actually decreased upon differentiation (in both cell lines used). We attribute this effect not to weakened antioxidant defence, but to directly increased paraquat toxicity by enhanced redox cycling (due to increased metabolic activity and availability of relatively more electron donors in differentiated cells), leading to excess reactive oxidant challenge at the same paraquat concentration. Such an effect has previously been documented in the literature [36].

When attempting to identify the molecular background of the recorded phenomenological effect of increased resistance to oxidants, we divided the potential mechanisms into two commonly described groups of antioxidants (“antioxidant systems”): low-molecular-weight thiols (GSH and thioredoxin) with their corresponding enzymatic synthesis and reduction machinery (since most of these enzymes are specific for one or the other of the thiols, we analysed them separately); and direct enzymatic scavengers for reactive oxygen species. Unsurprisingly, our results confirm that both of these systems have important functions in increased antioxidant activity during differentiation along the monocyte-macrophage axis. Differentiation (especially in the THP-1 early-stage model) caused induction of genes for proteins involved in all systems, notably of γ-glutamylcysteine ligase, thioredoxin reductase and manganese superoxide dismutase. Many (though not all) of these results could be confirmed by biochemical assays: Western blotting (e.g. induction of SOD2), enzymatic activity assays (e.g. induction of thioredoxin reductase) and antioxidant content (e.g. increase in GSH synthesis). Moreover, the application of specific pharmacological inhibitors (to thwart the resistance of differentiated cells) confirmed the importance i.a. of glutathione synthesis and superoxide dismutase activity for resistance of the less-mature THP-1 cells. We conclude that early stage differentiation is when overall mobilisation of cellular antioxidant defence capacity occurs, with strong contributions from thiol and non-thiol related antioxidant systems, with crucial roles of increased rate of glutathione synthesis as well as increased activity of manganese superoxide dismutase. Similar partial observations were made before with regard to thiol-related systems, such as transcriptional induction of thioredoxin and its reductase in differentiating THP-1 cells [34] or increased glutathione peroxidase activity in HL-60 cells differentiated towards a monocyte-like phenotype [37]. Similarly, increased expression and activity of superoxide dismutase, especially Mn-SOD, was reported upon differentiation of *ex vivo*-derived monocytes [38], U937 cells [39] and THP-1 cells [40]. It is important to note that our cellular models are relevant to early- and late-stage macrophage differentiation from monocytes, but are orthogonal to further cell fate polarisation (MI/MII macrophage types), so the late effects discussed here take place at a stage corresponding to physiological pre-polarisation events.

In view of recent reports of tight interplay between calcium and redox homeostasis in human monocytes [33], we verified the expression levels of regulators of redox sensitivity of store-operated calcium entry, the ORAI channels: ORAI1 (blocked by oxidation) and ORAI3 (oxidation-insensitive). In our study, the expression level of the *ORAI3* gene itself was a better predictor of oxidative stress resistance than *ORAI3*/*ORAI1* ratio, especially in the case of THP-1 cells where *ORAI1* was significantly induced by TLR2 signalling. However, underscoring the more monocyte-like character of Mono Mac 6 cells, in these cells we did observe a channel type transition similar to that seen for primary human monocytes [33], with both *ORAI3* level and *ORAI3*/*ORAI1* ratio increasing strongly after pam3CSK4 challenge, with the effect being strikingly synergistic with Mono Mac 6 differentiation. Thus, we can postulate that calcium homeostasis upon oxidative challenge should also be impacted in our cellular models, confirming the emerging consensus on the cross-talk between these two cellular events.

The most important and innovative part of our study was verification of the hypothesis that physiologically relevant signals modulating monocyte/macrophage function (in our case, activation of the pattern recognition receptor TLR2 as a canonical example of pathogen-triggered signalling) can contribute to antioxidant defence development. This hypothesis has been formulated in immunological literature based on two theoretical premises: on one hand, activation of monocytes and macrophages involves some elements in which redox homeostasis plays a major role (oxidative burst, phagocytosis, cytokine production and exocytosis); on the other hand, activation usually occurs in infected tissue microenvironments which are already oxidatively challenged due to inflammatory reaction, especially recruitment and activation of granulocytes [41–44]. There are few reports which directly verify this hypothesis with regard to TLR2—it has been shown e.g. that in mouse kidney ischaemia/reperfusion injury model, TLR2 is crucial to ERK1/2 activation in oxidative stress conditions [45].

Our results comprehensively support the theory of PRR involvement: activation of TLR2 contributed to antioxidant defence in cells with less and more advanced differentiation processes, modulating and fine-tuning molecular defence mechanisms and enhancing overall survival. This additional advantage from PRR activation for oxidative stress resistance was especially prominent for THP-1 cells, where a seemingly synergistic effect of differentiation and TLR2 activation was seen for cell survival, oxidative damage and several mechanistic effects (e.g. for thioredoxin reductase *TXNRD1* and thioredoxin peroxidase *PRDX1* genes as well as SOD2 gene expression, protein level and activity). However, also in more differentiated Mono Mac 6 cells, where further differentiation towards a more macrophage-like phenotype caused little (if any) advantage in terms of cellular resistance to oxidants and cellular level of antioxidants, sensitivity to TLR2 was retained. TLR2 activation in macrophage-like Mono Mac 6 conspicuously increased resistance to hydrogen peroxide and hydroxyl radical challenge as well as expression and activity of selected enzymes (most significantly thioredoxin reductase and both superoxide dismutases). While we cannot at this point identify the specific molecular pathway responsible for the observed effect of TLR2 activation, it is worth noting that both studied cell lines express a number of genes from the NFκB transcription factor family (Supplementary Table 2). This pathway is a known downstream effector of TLR2 [23, 24] and is often involved in regulation of stress response.

It is known that many of genes identified here as important for monocyte/macrophage redox homeostasis are under transcriptional control of the redox-sensitive transcription factor Nrf2 [46]. While oxidant regulation of Nrf2 activity proceeds mainly via its release from the KEAP1 cytoskeleton-associated protein and subsequent nuclear translocation, another well-known mechanism of its induction occurs through inhibition of its ubiquitination and consequent decrease of degradation/turnover and increase in cytoplasmic and nuclear levels [47]. This is exactly the phenomenon observed by us in THP-1 cells treated with TLR2 agonist—fast onset (seen after 1 h) and quick return to original levels prove that TLR2 activation stabilizes Nrf2 protein in the cytoplasm and allows efficient nuclear translocation by inhibiting its proteasomal degradation. It is interesting to observe that this increase is notably weaker in differentiated (more macrophage-like) THP-1 cells, while on the other hand these cells have higher nuclear levels of Nrf2 to start with. Consequently, TLR2 activation in undifferentiated and differentiated THP-1 cells leads to Nrf2 reaching similar absolute nuclear levels (since the nucleus is Nrf2's site of action, these are the levels relevant to its effect on gene expression), which may explain the lack of synergistic effects of differentiation and TLR2 activation for some of antioxidant genes studied. For example gene expression levels of *GCLM* and *PRDX6* after pam3CSK4 stimulation of differentiated THP-1 cells are no higher than in undifferentiated stimulated cells (for *GCLM*) or differentiated cells without stimulation (for *PRDX6*). It should be noted that we were unable to document a similar phenomenon in the Mono Mac 6 cell line due to extremely low basal expression of Nrf2 in these cells. This finding may contribute to the explanation of some surprising differences between cell lines in terms of gene expression, e.g. the *SOD1* gene, a well-known Nrf2 target, is induced by pam3CSK4 in THP-1 cells but not in Mono Mac 6 cells; *PRDX1*, also known to be strongly regulated by Nrf2 [48], also increases its expression only upon THP-1 differentiation and/or TLR2 activation, suggesting a transcriptional involvement of the Nrf2 pathway.

Thus, the bulk of our results support the notions that differentiation along the monocyte-macrophage axis, as well as pattern recognition receptor stimulation (exemplified by TLR2), causes a general shift in cellular redox homeostasis leading to overall stronger oxidative stress resistance. While we provide experimental confirmation of enhanced anti-oxidative protection both at the phenomenological level (cell viability, molecular oxidative damage) and regarding specific mechanisms (expression of genes, activity of enzymes), in models of differentiation and of TLR2 activation, we don't claim that all mechanisms and all effects are activated in both cell lines by both types of challenge. We rather suggest that all of our data, showing statistically significant differences in some cases and no difference in others, points to a general process (and specific genes/proteins involved in it) of gradual biochemical adaptation rather than a clear-cut qualitative leap in resistance. We have to bear in mind that our data were collected in *in vitro* cell line models—although they were very well characterised and commonly used ones, and we compared each effect carefully to the relevant control. Still, the relevance of the described phenomena to the physiological macrophage development *in vivo*, while highly probable, must be confirmed in further experiments, e.g. in laboratory animals or in actual human cells isolated *ex vivo*. This is a line of inquiry we are now pursuing.

## MATERIALS AND METHODS

### Cellular models

The THP-1 human acute monocytic leukemia cell line (ATCC, Rockville, MD, USA) was cultured in RPMI 1640 medium (Thermo Fisher Scientific, Carlsbad, CA, USA) supplemented with 10% fetal bovine serum (GE Life Sciences, Piscataway, NJ, USA), 1 mM sodium pyruvate (PAA), 100 U/ml penicillin (Thermo Fisher Scientific), 100 μg/ml streptomycin (Thermo Fisher Scientific) at 37°C in a humidified 5% CO_2_ incubator. The Mono Mac 6 human acute monocytic leukemia cell line (DSMZ, Braunschweig, Germany) was cultured in RPMI 1640 medium supplemented with 10% fetal bovine serum (GE Life Sciences), 1x Non-Essential Amino Acids (Invitrogen), 1x OPI Media Supplement (1 mM oxaloacetate, 0.45 mM pyruvate, 0.2 U/ml insulin, Sigma-Aldrich, St. Louis, MO, USA) at 37°C in a humidified 5% CO_2_ incubator. For differentiation, THP-1 cells were incubated in the presence of 20 ng/ml PMA (phorbol myristyl acetate, Sigma-Aldrich) for 72 hours. Mono Mac 6 cells were differentiated using 50 nM Vitamin D3 (Merck Millipore, Darmstadt, Germany) and 2 ng/ml TGFβ1 (R&D, Minneapolis, MN, USA) for 72 hours. Successful differentiation was confirmed by flow cytometry analysis of macrophage markers (see below). Undifferentiated and differentiated cells were stimulated with pam3CSK4 (In*vivo*gen, Toulouse, France) at a concentration of 500 ng/ml for 6 or 24 hours. In some experiments, TLR2 was blocked by pre-incubation with anti-TLR2 inhibitory antibody (PAb-hTLR2, In*vivo*gen) at a concentration of 5 μg/ml for 10 minutes. It was verified that TLR2 inhibition by antibody alone had no effect on antioxidant gene expression (data not shown).

### Flow cytometry

For expression analysis of macrophage marker surface proteins and TLR2, undifferentiated and differentiated as well as unstimulated and/or pam3CSK4-stimulated cells were resuspended in Flow Cytometry Staining Buffer (eBioscience, San Diego, CA, USA). To block Fc receptors and prevent nonspecific binding of Fc fragments, cells were incubated with Human Fc Receptor Binding Inhibitor Purified (eBioscience). 1 × 10^6^ cells were stained with anti-TLR2-FITC (mouse monoclonal, FAB2616F, R&D); anti-CD11b-FITC (mouse monoclonal, 11-0113, eBioscience); anti-CD11b-APC (mouse monoclonal, 17-0118, eBioscience); anti-CD14-PerCP-Cy5.5 (mouse monoclonal, 45-0149, eBioscience) or with appropriate isotype controls. Flow cytometric analysis was performed on LSRFortessa (BD Biosciences, San Jose, CA, USA) instrument and analyzed using FlowJo software (Tree Star Inc.). Since these experiments were performed for quality control reasons, to demonstrate the proper molecular characteristics of our cellular models, we do not include these results as figures in the results section. Representative flow cytometry histograms may be found in supplementary material as Supplementary Figure 1 (differentiation markers) and Supplementary Figure 2 (TLR2 expression).

### Cell viability assay

For viability assays, undifferentiated and differentiated as well as unstimulated and pam3CSK4-stimulated cells were seeded at 3.5 × 10^5^ per well in a 96-well plate and incubated with increasing reactive oxidant concentrations for 6 h. The following reactive oxidants were used: hydrogen peroxide (Sigma-Aldrich), a 1:10 (molar) mix of ferrous chloride (Sigma-Aldrich) and ascorbic acid (Sigma-Aldrich), and paraquat (Sigma-Aldrich). In some experiments, cells were pre-incubated overnight with buthionine sulfoximine (GSH synthesis inhibitor, Sigma-Aldrich), carmustine (glutathione reductase inhibitor, Sigma-Aldrich), auranofin (thioredoxin reductase inhibitor, Enzo Life Sciences, Farmingdale, NY, USA), diethyldithiocarbamate (SOD inhibitor, Sigma-Aldrich) or sodium azide (catalase inhibitor, Sigma-Aldrich) and subsequently incubated with hydrogen peroxide.

Cell viability was assessed using a resazurin-based method [49]. Cells were rinsed with Phosphate-Buffered Saline (PBS, Thermo Fisher Scientific) and subsequently incubated in 0.0125 mg/ml resazurin (Sigma-Aldrich) solution in Hanks's Balanced Salt Solution (Thermo Fisher Scientific) with 1 mg/ml glucose (Sigma-Aldrich) in 96-well microplates. Plates were incubated for 1 h at 37°C in darkness. Fluorescence intensity of reduced resorufin was measured using EnVision Multilabel Plate Reader (PerkinElmer, Waltham, MA, USA) at 530 nm excitation and 590 nm emission wavelength.

### Protein oxidative damage assay

For oxidative damage assays, undifferentiated and differentiated as well as unstimulated and pam3CSK4-stimulated cells were treated with 1 mM hydrogen peroxide or a mixture of 1 mM ferrous chloride/10mM ascorbic acid for 2 h. Cell lysate was prepared using RIPA buffer (Sigma-Aldrich) and incubated with equal amount of 0.2 mM fluorescein-5-thiosemicarbazide (Thermo Fisher Scientific) in Hanks's Balanced Salt Solution (Thermo Fisher Scientific) overnight in darkness [50]. Four volumes of ice-cold 20% trichloroacetic acid (Sigma-Aldrich) was subsequently added, the sample was incubated for 10 minutes on ice and centrifuged. The pellet was washed three times with acetone and air-dried. Samples were solubilised in 6 M guanidine hydrochloride (Sigma-Aldrich) and diluted 10 times with 0.1 M monosodium phosphate (Sigma-Aldrich). Samples were transferred to a 96-well black microplate and fluorescence was measured at 485 nm excitation and 535 nm emission wavelength. Protein concentration was determined using the Pierce BCA Protein Assay kit (Thermo Fisher Scientific).

### Gene expression assay

Total cellular RNA was isolated using TRI Reagent (Sigma-Aldrich) according to manufacturer's protocol. Complementary DNA was synthesized using Maxima First Strand cDNA Synthesis Kit for RT-qPCR (Thermo Fisher Scientific) with 4 μg of RNA used per reaction. PCR amplification was performed in 384-well microplates at 10 μl of total volume, containing cDNA, 0.5 μM primer mix (primer sequences in Supplementary Table 1) and LightCycler 480 SYBR Green I Master Mix (Roche Applied Science, Penzberg, Germany). Real time PCR amplifications were performed in a LightCycler 480 (Roche) under the following conditions: 40 cycles of denaturation at 95°C for 10 s, annealing at 60°C for 10 s, and extension at 72°C for 20 s. Three reference (constitutive expression) genes were selected using the GeNorm procedure [51]: *HPRT1*, *HMBS* and *TBP*. Expression of assayed genes was expressed as number of cognate mRNA copies per 1000 copies of geometric-averaged reference mRNA.

### Determination of glutathione content

For glutathione assays, undifferentiated and differentiated as well as unstimulated and pam3CSK4-stimulated cells were washed twice with PBS and lysed for 5 minutes in a solution containing 20 mM HCl, 5 mM diethylene triamine pentaacetic acid (Sigma-Aldrich), 10 mM ascorbic acid and 5% trichloroacetic acid (Sigma-Aldrich). The suspension was centrifuged at 12,000 g, the supernatant was neutralised with 1 M potassium phosphate buffer (pH 7.0) and divided into two aliquots, one of which was reacted with 0.45 mM N-ethylmaleimide (Sigma-Aldrich). Both samples were subsequently diluted with 0.1 M potassium phosphate buffer (pH 7.0) and reacted with 0.05% o-phthalaldehyde (Sigma-Aldrich). Samples were incubated for 30 min at RT and fluorescence was measured using EnVision Multilabel Plate Reader at an excitation wavelength of 355 nm and an emission wavelength of 430 nm. Reduced glutathione content in a sample was expressed as difference between fluorescence values in non-derivatised and N-ethylmaleimide-derivatised sample aliquots.

### Western blotting

For Western blotting, undifferentiated and differentiated as well as unstimulated and pam3CSK4-stimulated cells were washed with ice-cold PBS and lysed in ice-cold RIPA buffer (Sigma-Aldrich) with the addition of Halt Protease Inhibitor Cocktail (Thermo Fisher Scientific) for 30 minutes at 4°C with constant agitation. The lysate was centrifuged for 15 min at 10,000 g. Nuclear and cytoplasmic proteins were extracted using NE-PER Nuclear and Cytoplasmic Extraction Reagents (Thermo Fisher Scientific). Protein concentration in the supernatant was determined using the Pierce BCA Protein Assay kit (Thermo Fisher Scientific). An aliquot containing 25 μg of protein was mixed with NuPage Reducing Agent (Thermo Fisher Scientific) and NuPage Sample Buffer (Thermo Fisher Scientific), incubated for 10 min at 70°C and electrophoretically separated on 12% Bis–Tris NuPage precast gel (Thermo Fisher Scientific). Proteins were transferred onto Hybond-C membrane (GE Life Sciences) using the iBlot dry blotting system (Thermo Fisher Scientific). Membranes were blocked with 20% milk and immunostained with primary antibodies followed by HRP-conjugated secondary antibody (Abcam, Cambridge, UK). The following antibodies were used: anti-thioredoxin (rabbit monoclonal, EPR6110, Abcam); anti-thioredoxin reductase 1 (rabbit polyclonal, ab16840, Abcam); anti-peroxiredoxin 1 (rabbit monoclonal, EPR5433, Abcam); anti-superoxide dismutase 2 (rabbit polyclonal, ab13533, Abcam); anti-Nrf2 (rabbit monoclonal, ab62352, Abcam); anti-β-actin (rabbit polyclonal, ab8227, Abcam); anti-TBP (mouse monoclonal, ab51841, Abcam). Specific bands were visualized using SuperSignal West Pico Chemiluminescent Substrate (Thermo Fisher Scientific) and G-Box chemiluminescence imaging station (Syngene, Cambridge, UK). Signal strength in cognate bands corresponding to assayed proteins was quantified with G-Box station software and standardised with reference to signal strength of the β-actin band.

### Antioxidant enzyme assays

For enzyme assays, undifferentiated and differentiated as well as unstimulated and pam3CSK4-stimulated cells were washed twice with PBS.

Glutathione reductase activity was measured by monitoring the rate of NADPH oxidation [52]. 1 × 10^5^ cells were lysed in 50 μl of 100 mM potassium phosphate buffer (pH 7.5) containing 1 mM EDTA and 1% Triton X-100. Cell lysate was mixed with 50 μl of 4 mM GSSG solution and 100 μl of 200 μM NADPH solution (Sigma-Aldrich). Oxidation of NADPH was monitored spectrophotometrically in kinetic mode for 5 minutes at 340 nm. Glutathione reductase activity was proportional to the rate of absorbance decrease.

Glutathione peroxidase activity was measured indirectly by monitoring NADPH consumption in a coupled reaction with glutathione reductase [53]. 1 × 10^5^ cells were lysed in 50 μl of 50 mM Tris-HCl buffer (pH 8.0) containing 0.5 mM EDTA and 0.1% Triton X-100. Cell lysate was mixed with 50 μl of solution containing 1 mM NADPH, 8.5 mM GSH, 2 U/ml GR (Sigma-Aldrich). Reaction was initiated by adding 100 μl of 0.6 mM tert-butyl hydroperoxide (Sigma-Aldrich) solution. Oxidation of NADPH, coupled with oxidized glutathione reduction, was monitored spectrophotometrically in kinetic mode for 5 minutes at 340 nm. Glutathione peroxidase activity was proportional to the rate of absorbance decrease.

Thioredoxin reductase activity was measured by monitoring the conversion of DTNB to TNB by reduced thiols [53]. 1×10^5^ cells were lysed in 50 μl of 100 mM potassium phosphate buffer (pH 7.5) containing 1 mM EDTA and 1% Triton X-100. Another aliquot of the same cell sample was lysed in the same lysis buffer, but containing additionally 3 μM auranofin as thioredoxin reductase inhibitor. Cell lysate was mixed with 100 μl of solution containing 5 mM DTNB and 250 μM NADPH. Absorbance was monitored spectrophotometrically in kinetic mode for 5 minutes at 405 nm. Thioredoxin reductase activity was proportional to the difference in TNB generation rate in samples lysed without and with auranofin.

Catalase activity was assayed by monitoring the rate of removal of exogenously added hydrogen peroxide in a colorimetric reaction with Trinder's reagent [54]. 1 × 10^5^ cells were lysed in 50 μl of 50 mM potassium phosphate buffer (pH 7.0) containing 0.1% Triton X-100, and incubated for 3 minutes with 50 mM hydrogen peroxide. The catalase reaction was stopped by adding sodium azide to 15 mM final concentration and the remaining hydrogen peroxide was assayed in a reaction with Trinder's reagent (final concentrations 0.225 mM 4-aminoantipyrine, 1.8 mM 3,5-dichloro-2-hydroxybenzenosulfonic acid, 0.675 U/ml | horseradish peroxidase; Sigma–Aldrich) for 30 min. Absorbance was measured at 520 nm. Catalase activity was proportional to the difference in absorbance between a control sample (without cell lysate) and the assayed sample.

Superoxide dismutase activity was assayed by monitoring the rate of removal of exogenously added superoxide (from redox cycling of phenazine methosulfate with NADH) in a colorimetric reaction with nitro blue tetrazolium [55]. 1 × 10^5^ cells were lysed in 50 μl of 10 mM potassium phosphate buffer (pH 7.4) containing 0.1 mM EDTA and 10 μM diphenylene iodonium (to inhibit endogenous superoxide generation), for 1 minute in an ultrasound bath. Cell lysate was extracted with ethanol/chloroform at 20% and 11% final concentrations, respectively, to remove proteins other than SOD which might have dismutase activity. 50 μl of aqueous phase was mixed with 100 μl of solution containing 160 μM NADH and 100 μM nitro blue tetrazolium (Sigma-Aldrich). 50 μl of 13 μM phenazine methosulfate (Sigma-Aldrich) solution was added and the change in absorbance was monitored in kinetic mode for 5 minutes at 560 nm. Superoxide dismutase activity was proportional to the difference in absorbance increase rate between a control sample (without cell lysate) and the assayed sample.

EnVision Multilabel Plate Reader was used to measure absorbance in all cases.

### Statistics

For statistical significance testing we used one-way ANOVA for concentration series followed by post-hoc Tukey's test for pairwise difference testing. For single pairwise comparisons (e.g. real-time PCR results), Student's *t* test was applied. In all tests, *p* values < 0.05 were considered to be statistically significant. IC50 values for viability data were derived by sigmoidal (logistic) regression using the Graphpad Prism software package. Data are presented as arithmetic mean ± S.E.M. where appropriate, except for real-time PCR results where due to exponential transformation during calculations, data are presented as geometric means with transformed upper S.E.M. values.

## SUPPLEMENTARY MATERIALS FIGURES AND TABLES


