# Supplementary material for: TLR2 activation induces antioxidant defence in human monocyte-macrophage cell line models
Source: Oncotarget. 2017 Apr 21;8(33):54243–64. doi: 10.18632/oncotarget.17342 (PMC5589577; doi:10.18632/oncotarget.17342)
Supplement: Supplementary file 1 [file oncotarget-08-54243-s001.pdf]

TLR2 activation induces antioxidant defence in human monocyte-macrophage cell line models

Supplementary Materials

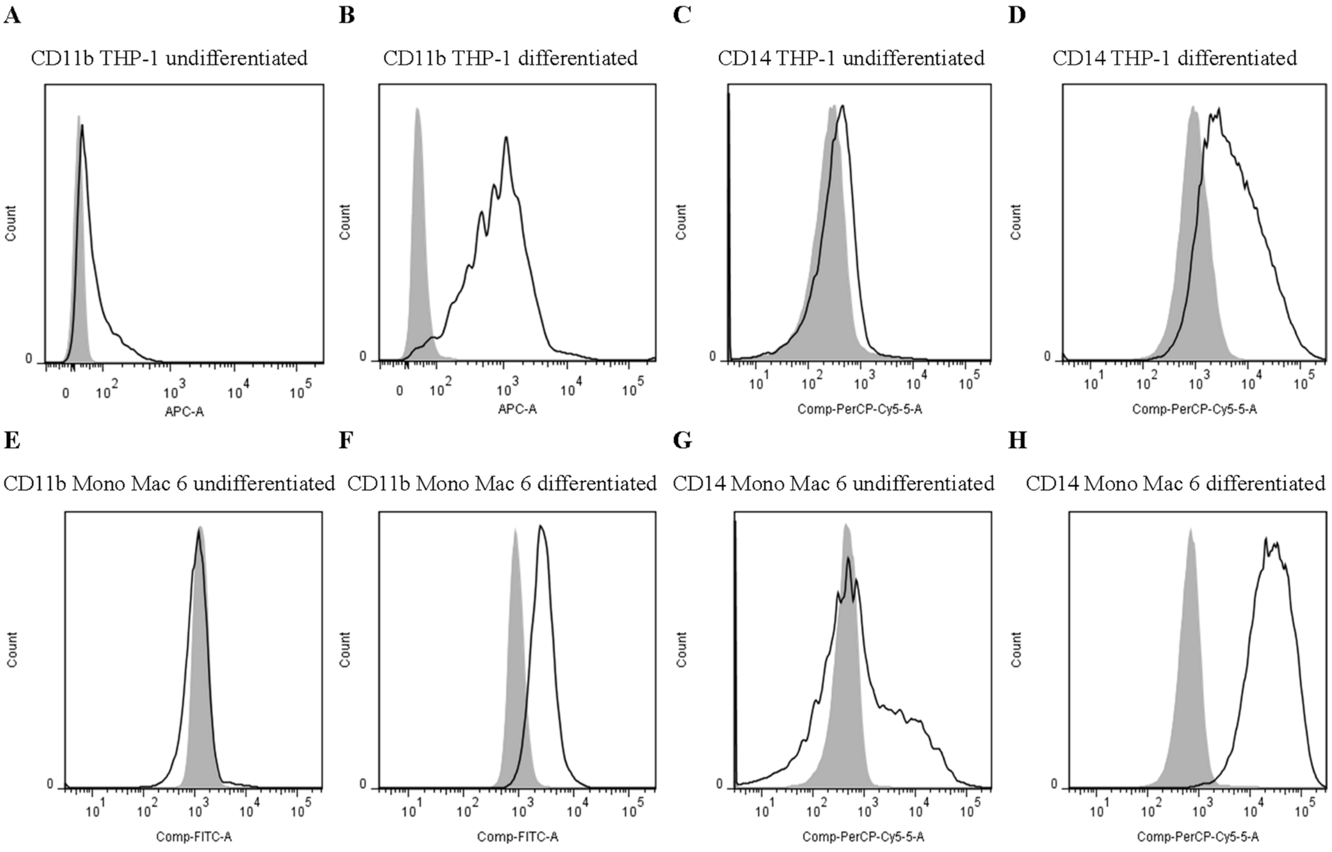

Supplementary Figure 1: Differentiation protocols induce macrophage marker expression in THP-1 and Mono Mac 6 cell lines. THP-1 (**A**–**D**) and Mono Mac 6 (**E**–**H**) cells were assayed without differentiation (A, C, E, G) or after differentiation (B, D, F, H) as described in Materials and methods. Subsequently, surface protein expression of CD11b (A, B, E, F) and CD14 (C, D, G, H) was assayed by flow cytometry with respective antibodies as described in Materials and methods. Data presented as representative flow cytometry histograms with unshaded histogram corresponding to the cognate antibody and shaded histogram – to the relevant isotype control.

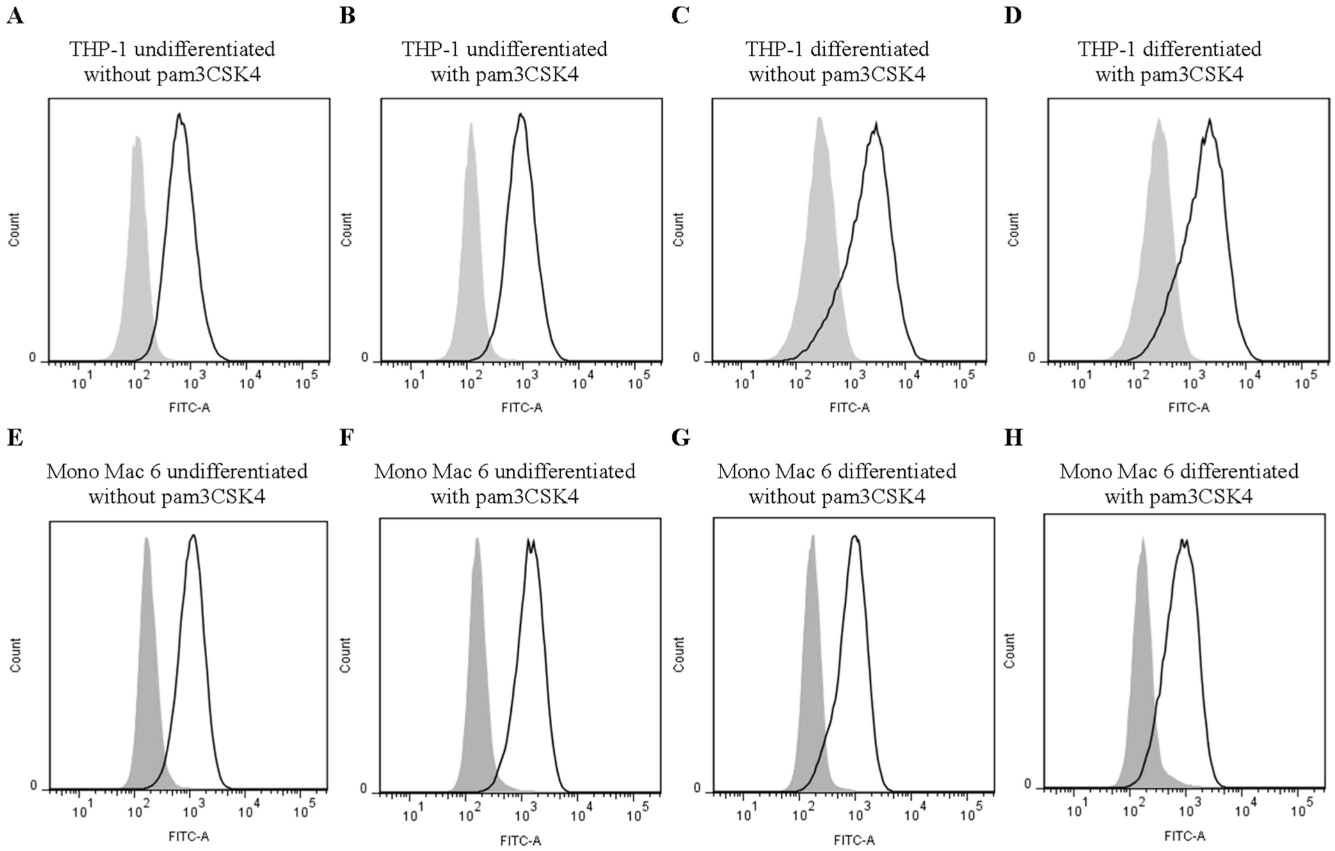

Supplementary Figure 2: TLR2 is expressed on the cell surface of THP-1 and Mono Mac 6 cell lines before and after differentiation and ligand stimulation. THP-1 (**A**–**D**) and Mono Mac 6 (**E**–**H**) cells were assayed without differentiation (A, B, E, F) or after differentiation (C, D, G, H), without pam3CSK4 treatment (A, C, E, G) or after 500 ng/ml pam3CSK4 treatment for 24 h (B, D, F, H), as described in Materials and methods. Subsequently, surface protein expression of TLR2 was assayed by flow cytometry with anti-TLR2 antibody as described in Materials and methods. Data presented as representative flow cytometry histograms with unshaded histogram corresponding to the cognate antibody and shaded histogram – to the isotype control.

Supplementary Table 1: PCR primers used in real-time RT-PCR gene expression assays

Gene	Forward and reverse sequences (5′–3′)	Product size (bp)	Source
HPRT1	Fw: TGACACTGGCAAAACAATGCA<br>Rv: GGTCCTTTTCACCAGCAAGCT	94	RTPrimerDB, ID 5
HMBS	Fw: GGCAATGCGGCTGCAA<br>Rv: GGGTACCCACGCGAATCAC	64	RTPrimerDB, ID 4
TBP	Fw: CACGAACCACGGCACTGATT<br>Rv: TTTTCTTGCTGCCAGTCTGGAC	89	RTPrimerDB, ID 8098
GCLM	Fw: CATTTACAGCCTTACTGGGAGG<br>Rv: CGTGCGCTTGAATGTCAGG	298	PrimerBank
GCLC	Fw: GGCACAAGGACGTTCTCAAGT<br>Rv: CAAAGGGTAGGATGGTTTGGG	160	PrimerBank
GPX1	Fw: TTCCCGTGCAACCAGTTTG<br>Rv: TTCACCTCGCACTTCTCGAA	128	QPPD
GPX2	Fw: AATGTGGCTTCGCTCTGAGG<br>Rv: GTCCTTCAGGTAGGCGAAGAC	261	PrimerBank
GPX3	Fw: TTGATGGGGAGGAGTACATCC<br>Rv: AGACCGAATGGTGCAAGCTC	139	PrimerBank
GPX4	Fw: CGGGCTACAACGTCAAATTCG<br>Rv: GGGGCAGGTCCTTCTCTATCA	222	PrimerBank
GPX7	Fw: TGTTTAGCAAGATTGCAGTCACC<br>Rv: TTTCCATCTGGGGCTACTAGG	121	PrimerBank
GSS	Fw: CCCTGGCTGAGGGAGTATTG<br>Rv: TGCACAGCATAGGCTTGCTC	130	PrimerBank
GLRX	Fw: CCAACCACACTAACGAGATTCA<br>Rv: TCACTGCATCCGCCTATACAA	103	PrimerBank
PRDX6	Fw: GTTGCCACCCCAGTTGATTG<br>Rv: TGAAGACTCCTTTCGGGAAAAGT	100	PrimerBank
TXN	Fw: CAACCCTTTCTTTCATTCCCTCT<br>Rv: CACCCACCTTTTGTCCCTTCT	149	PrimerBank
TXN2	Fw: TCAAGACCGAGTGGTCAACAG<br>Rv: CACCTCATACTCAATGGCGAG	181	PrimerBank
TXNRD1	Fw: TCCTATGTCGCTTTGGAGTGC<br>Rv: GGACCTAACCATAACAGTGACGC	72	QPPD
TXNRD2	Fw: GGACTACGTGGAACCTTCTCC<br>Rv: AACCGTGTGCTCGTCAACAAA	298	PrimerBank
PRDX1	Fw: AATGCTAAAATTGGGCACCCT<br>Rv: TGAAAGCAATGATCTCCGTGG	166	PrimerBank
PRDX3	Fw: GCAGATTTCCCGAGACTACG<br>Rv: GCTCAAATGCTTGATGACTCC	100	PrimerBank
SESN1	Fw: CAGCATTGGAAAACATTAGGCAA<br>Rv: CCGAAGACTCGGTATTTGAAAGC	111	PrimerBank
SOD1	Fw: AGGGCATCATCAATTTCGAG<br>Rv: TGCCTCTCTTCATCCTTTGG	196	QPPD
SOD2	Fw: AACCTCAGCCCTAACGGTG<br>Rv: AGCAGCAATTTGTAAGTGTCCC	180	PrimerBank
CAT	Fw: ACTTTGAGGTCACACATGACATT<br>Rv: CTGAACCCGATTCTCCAGCA	117	PrimerBank
ORAI1	Fw: ATGAGCCTCAACGAGCACT<br>Rv: GTGGGTAGTCGTGGTCAG	190	[33]
ORAI3	Fw: GTACCGGGAGTTCGTGCA<br>Rv: GGTACTCGTGGTCACTCT	192	[33]
REL	Fw: TGCTCTTCCTCCTGTTGTCTCG<br>Rv: TCTGCAGTATTTGGAGCACGGTTG	60	QuantPrime
RELA	Fw: TATCAGTCAGCGCATCCAGACC<br>Rv: CGCTGCTCTTCTATAGGAACTTGG	60	QuantPrime
RELB	Fw: AGGAGATTGAGGCTGCCATTGAG<br>Rv: TTCTTCAGGGACCCAGCGTTGTAG	73	QuantPrime
NFKB1	Fw: CCTCCACAAGGCAGCAAATAGACG<br>Rv: AGCTGAGTTTGCGGAAGGATGTC	87	QuantPrime
NFKB2	Fw: ACTCCTCCATTGTGGAACCCAAG<br>Rv: TCGAAATCGGAAGCCTCTCTGC	101	QuantPrime

For each primer pair, gene name, primer sequences, amplicon size and source of primer sequence is given. RTPrimerDB is a public database available at the University of Gent, Belgium (www.rtprimerdb.org; [39]). PrimerBank is a public database available at the Harvard University, USA (pga.mgh.harvard.edu/primerbank; [3]). QPPD (Quantitative PCR Primer Database) is a public database available at the National Cancer Institute, USA (lpgws.nci.nih.gov/cgi-bin/PrimerViewer). QuantPrime is a primer design tool (www.quantprime.de; [57]).

Supplementary Table 2: NF-κB gene expression levels in monocyte/macrophage cell lines

	**THP-1**				**Mono Mac 6**			
**Gene**	**Undifferentiated without pam3CSK4**	**Undifferentiated with pam3CSK4**	**Differentiated without pam3CSK4**	**Differentiated with pam3CSK4**	**Undifferentiated without pam3CSK4**	**Undifferentiated with pam3CSK4**	**Differentiated without pam3CSK4**	**Differentiated with pam3CSK4**
REL	830/45<br>(**100**)	2448[#]/89<br>(**300**)	935/62<br>(**110**)	6557[#]/902<br>(**790**)	834/98<br>(**100**)	1575[#]/261<br>(**190**)	4174[*]/1657<br>(**500**)	11057/3914<br>(**1330**)
RELA	2350/116<br>(**100**)	3085[#]/113<br>(**130**)	5423[*]/636<br>(**230**)	7216/558<br>(**310**)	1998/58<br>(**100**)	2448[#]/74<br>(**120**)	3221/689<br>(**160**)	4856/1501<br>(**240**)
RELB	414/45<br>(**100**)	1793[#]/20<br>(**430**)	2202[*]/182<br>(**530**)	3321[#]/120<br>(**800**)	188/34<br>(**100**)	1350[#]/105<br>(**720**)	149/1<br>(**80**)	876[#]/70<br>(**470**)
NFKB1	2044/231<br>(**100**)	11035[#]/320<br>(**540**)	4709[*]/444<br>(**230**)	11980[#]/884<br>(**590**)	2714/52<br>(**100**)	8128[#]/634<br>(**300**)	2230/117<br>(**80**)	9999[#]/623<br>(**370**)
NFKB2	708/114<br>(**100**)	8524[#]/379<br>(**1200**)	5798[*]/617<br>(**820**)	19860[#]/1706<br>(**2800**)	540/28<br>(**100**)	5397[#]/382<br>(**1000**)	516/16<br>(**100**)	5113[#]/60<br>(**950**)

THP-1 and Mono Mac 6 cells were assayed without differentiation or after differentiation as described in Materials and methods. Undifferentiated and differentiated cells were subsequently untreated or treated with 500 ng/ml pam3CSK4 (for TLR2 simulation) for 6 h. Subsequently, gene expression was assayed at the mRNA level by the real-time RT-PCR method. Data expressed as relative mRNA copy number per 1000 copies of averaged reference mRNA, calculated by $2^{-\Delta Ct}$ transformation, presented as mean/transformed upper S.E.M. and separately (below, in parenthesis) as percentage of expression level in the undifferentiated/unstimulated sample; $n = 3$. Asterisks indicate statistically significant difference between the undifferentiated and differentiated cells, $p < 0.05$; hash signs indicate statistically significant difference between the TLR2-stimulated and unstimulated cells, $p < 0.05$.
